# Synthesis, Characterization, and Reaction of Digermylenes

**DOI:** 10.1002/asia.202200141

**Published:** 2022-05-16

**Authors:** Shuai‐Cong Huo, Yao Li, De‐Xiang Zhang, Qi Zhou, Ying Yang, Herbert W. Roesky

**Affiliations:** ^1^ School of Chemistry and Chemical Engineering Central South University Lushannan Road 932 410083 Changsha P. R. China; ^2^ Institut für Anorganische Chemie Georg-August-Universität Tammannstraβe 4 37077 Göttingen Germany

**Keywords:** Main group elements, Germanium, Germylene, Digermylene, Nucleophilic addition

## Abstract

A series of digermylenes R(EGeL)_2_ (L=CH[C(Me)N(Ar)]_2_, Ar=2,6‐*i*Pr_2_C_6_H_3_; E=O, R=1,3‐C_6_H_4_ (**1**), 1,4‐C_6_H_4_ (**2**), Me_2_C(CH_2_)_2_ (**3**); E=NH, R=1,4‐C_6_H_4_ (**4)**, 1,4‐C_6_H_10_ (**5**); E=C(O)O, R=1,3‐C_6_H_4_ (**6**)) were synthesized by the reactions of L′Ge (L′=HC[C(CH_2_)N(Ar)]C(Me)N(Ar), Ar=2,6‐*i*Pr_2_C_6_H_3_) with selected diphenols, diol, diamines, and *o*‐/*m*‐phthalic acids, respectively. Treatment of digermylene 1,3‐C_6_H_4_(OGeL)_2_ (**1**) with sulfur, selenium and CuX (X=Cl, Br, I) led to the formation of 1,3‐C_6_H_4_[OGe(S)L]_2_ (**8**), 1,3‐C_6_H_4_[OGe(Se)L]_2_ (**9**), and (CuX)_2_[1,3‐C_6_H_4_(OGeL)_2_]_2_ (X=Cl (**10**), Br (**11**), I (**12**)), respectively. The obtained products were characterized by melting point, elemental analysis, FT‐IR, ^1^H and ^13^C NMR spectroscopy, and single‐crystal X‐ray diffraction.

## Graphical abstract

**A series of digermylenes** R(EGeL)_2_ (L=CH[C(Me)N(Ar)]_2_, Ar=2,6‐*i*Pr_2_C_6_H_3_; E=O, R=1,3‐C_6_H_4_ (**1**), 1,4‐C_6_H_4_ (**2**), Me_2_C(CH_2_)_2_ (**3**); E=NH, R=1,4‐C_6_H_4_ (**4)**, 1,4‐C_6_H_10_, (**5**); E=C(O)O, R=1,3‐C_6_H_4_ (**6**)) were synthesized by the nucleophilic addition of diphenols, diol, diamines and dicarboxylic acids, respectively, with L′Ge (L′=HC[C(CH_2_)N(Ar)]C(Me)N(Ar), Ar=2,6‐*i*Pr_2_C_6_H_3_). Treatment of the obtained digermylene 1,3‐C_6_H_4_(OGeL) (**1**) with sulfur, selenium, and CuX (X=Cl, Br, I, C_6_F_6_) is also explored.

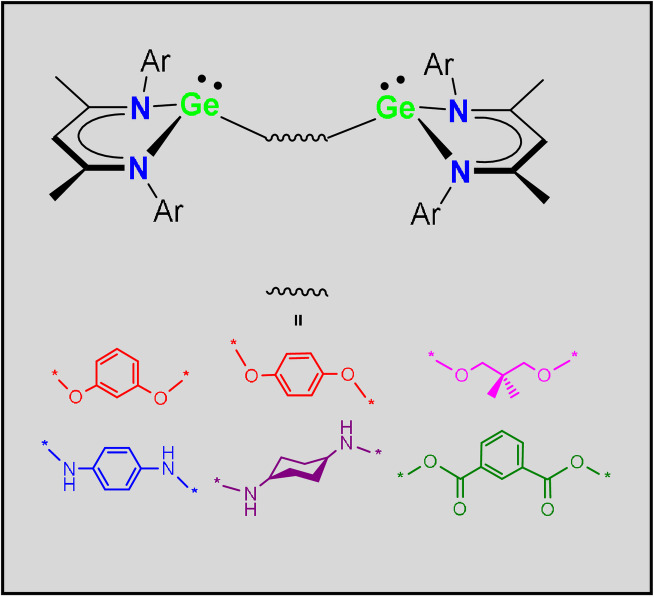

## Introduction

**Scheme 1:**
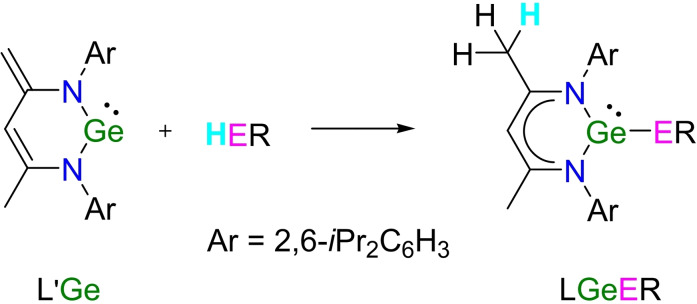
The reaction of L′Ge with HER (E=N,^[16]^ O,^[17]^ and P^[18]^).

In past decades, the stabilization and isolation of heavier analogues of carbenes have attracted widespread attention for their great importance in fundamental and applied chemistry.^[1]^ Parallel to the significant studies on the ditetrelenes (R_2_M=M′R_2_)^[2]^ and ditetrylynes,^[3]^ particularly digermynes featured with the multiple‐ or single‐bonded Ge(I)‐Ge(I) structure,^[4]^ the research on the digermylenes with more distant Ge(II)⋅⋅⋅Ge(II) separation has been extensively explored. One representative example is the digermylene oxide with the oxygen atom connecting the two Ge(II) centers.^[5]^ Greater spacing distances between two Ge(II) sites have been achieved by using *p*‐benzenenediamido,^[6]^ *p*‐phenylene, *p*‐biphenylene,^[7]^ *p*‐terphenylene,^[8]^ cyclohexylene,^[9]^ or flexible alkyl chains^[10]^ linkers in specially designed ligands. Intriguingly, the elimination of HCl from the *β*‐diketiminate ligand supported germylene chloride LGeCl (L=CH[C(Me)NAr]_2_, Ar=2,6‐*i*Pr_2_C_6_H_3_)^[11]^ gave a heterofulvene‐like germylene L′Ge (L′=HC[(C−CH_2_)CMe](NAr)_2_) (Scheme 1),^[12]^ which unexpectedly produced digermylenes by reactions with dibromoethane,^[12]^ HN(SiMe_3_)_2_,^[13]^ PhC(N*t*Bu)_2_GeCl,^[14]^ or (HO)_2_BPh.^[15]^ Furthermore, L′Ge has been found to show abundant reactivity with HER (E=N, O, P), forming LGeER through bond cleavage and nucleophilic addition of H−N,^[16]^ H−O,^[17]^ and H−P^[18]^ (Scheme 1). This strategy appears more convenient and attractive for the synthesis of digermylenes when considering the use of (HE)_2_R which contains two functional groups for the reaction with L′Ge. In a recent practice, we used (HO)_2_BR (R=2,4,6‐Me_3_C_6_H_2_, 1‐Naph) as the organoboryl source to treat L′Ge in a 1 : 2 ratio, precisely leading to the formation of digermylene RB(OGeL)_2_ as expected,^[19]^ while another parallel reaction with (HO)_2_BR (R=2,4‐Me_2_C_6_H_3_) only afforded RB(OH)OGeL, rather than a binuclear product. These findings suggest that the above approach is useful in the synthesis of digermylenes and also deserves more exploration when the effect of substituents is taken into account.

Herein, we report the synthesis of the digermylenes via the direct reaction of L′Ge with diphenols, diol, diamines, and *o*‐/*m*‐phthalic acids. The reactivity concerning the functional groups, spacer size, and substituent position are investigated. Furthermore, as known, germylenes show efficient Ge→Cu donation[^5c^, ^10f^, ^20^] and have oxidative addition reactivity with elemental chalcogen,^[21]^ allowing stabilization of new derivatives. Based on our synthetic work on germylenes^[22]^ and diacylthioureas,^[23]^ the reactivity studies on the obtained representative digermylene with sulfur, selenium, and CuX (X=Cl, Br, I, C_6_F_5_) are carried out. The resultant products were characterized by melting point, elemental analysis, FT‐IR, ^1^H and ^13^C NMR spectroscopy, and single‐crystal X‐ray diffraction.

## Results and Discussion

### The reaction of L′Ge with diphenols

**Scheme 2:**
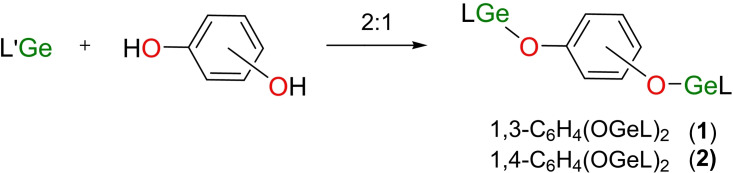
The reactions of L′Ge with *m*‐diphenol and *p*‐diphenol.

Previously, we have proved that the addition reaction of L′Ge with PhOH enabled the synthesis of the mononuclear germylene LGeOPh.^[17]^ To ascertain the applicability of the reaction of diphenol with L′Ge for the preparation of digermylene, the toluene solution of L′Ge was treated with *o*‐, *m*‐, and *p*‐diphenols in ether, respectively, in a 2 : 1 ratio (Scheme 2). After slowly mixing the reactant solutions at −78 °C and then stirring the reaction mixtures overnight at room temperature, volatiles were removed *in vacuo*, and ^1^H NMR spectra of C_6_D_6_ solutions of the residues were recorded. It was indicated that the reaction of L′Ge with *o*‐diphenol gave a complex mixture of products, involving the large presence of free ligand LH, which may be formed by the ligand exchange between L′ and *o*‐diphenol. This reaction, which did not follow the pre‐design, was mainly initialized by the bidentate nature of *o*‐diphenol. In contrast, the digermylene 1,3‐C_6_H_4_(OGeL)_2_ (**1**) was readily formed by the reaction of L′Ge with *m*‐diphenol, as yellowish solid powders in high yield (90%).

**Figure 1:**
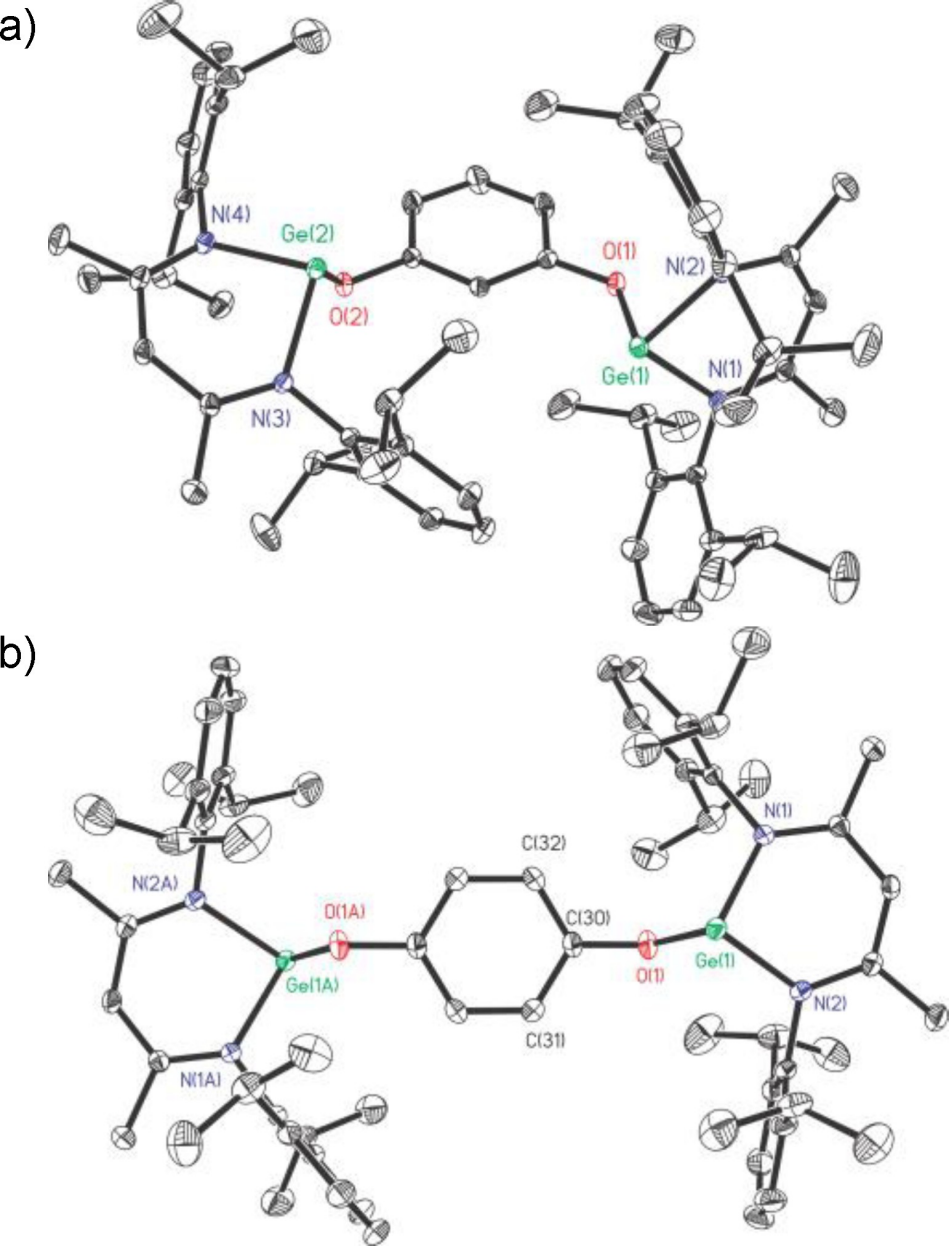
The molecular structures of **1** (a) and **2** (b) with the anisotropic displacement parameters are depicted at the 30% probability level. The H atoms are omitted for clarity. Selected bond lengths (Å) and angles (deg): a) for **1**, Ge(1)−O(1) 1.884(2), Ge(2)−O(2) 1.849(2); Ge(1)−O(1)−C(60) 125.4(2), Ge(2)−O(2)−C(64) 117.11(19); b) for **2**, Ge(1)−O(1) 1.8494(17); Ge(1)−O(1)−C(30) 116.09(14).

The melting range of **1** (271.3–273.8 °C) is much higher than that of LGeOPh (203 °C).^[17]^ The ^1^H NMR spectrum of **1** exhibits a characteristic singlet at *δ* 4.68 ppm, corresponding to the *γ*‐C*H* protons on the framework of *β*‐diketiminate ligands, while two septets at *δ* 3.10 and 3.48 ppm in a ratio of 1 : 1 are assignable to the methine protons of the isopropyl groups on the Ar groups. The sharp singlet at *δ* 1.60 ppm for the protons of methyl groups connected to the *β*‐C indicated the successful cleavage and nucleophilic addition of H−O moieties. It was suggested the two [LGe] moieties are equivalent in solution due to the conformational averaging by free rotation of σ bonds. Besides the presence of all the expected protons, the correct integral ratio confirmed the 2 : 1 reaction. Compound **1** has good solubility in toluene and tetrahydrofuran but is less soluble in *n*‐hexane. Pale yellow bulk crystals of **1** with a quality suitable for single‐crystal X‐ray diffraction characterization were obtained from the mixed solvent of toluene and hexane at −20 °C for one week. Compound **1** crystallizes in the triclinic *P*‐1 space group. As shown in Figure 1a, in the solid‐state the two [LGe] moieties adopted asymmetric arrangement. One C_3_N_2_ quasi‐plane is nearly perpendicular to the bridging phenyl plane, while the other is almost parallel to the latter. The C_3_N_2_Ge six‐membered rings both adopt the twisted boat conformation, where Ge atoms are located in the bow position and *γ*‐C atoms in the stern position. From the perspective of the O atoms, the Ge(1) boat is placed in a normal face‐up position and the other Ge(2) boat is placed upside down, suggesting the flexibility of the *β*‐diketiminate ligand by complying with the crystallization. The Ge−O bond lengths (1.884(2) Å and 1.849(2) Å) are within the reasonable range.^[24]^ The two Ge atoms are located on the same side of the molecule, with distances of 0.389 Å and 1.452 Å from the bridging phenyl plane, respectively (Figure S1). Besides, the Ge⋅⋅⋅Ge separation of **1** (5.668 Å) is much longer than those in MesB(OGeL)_2_ (Mes=2,4,6‐Me_3_C_6_H_2_) (3.682 Å)^[19]^ and digermylene oxides (3.1–3.2 Å),^[5c]^ while shorter than those in [LGe(OBPh)_2_]_2_O (9.555 Å) or [(LGeOBO)_2_O]BMes (6.962 Å).^[15]^ Based on our experience with diacylthioureas,^[23]^ this donor⋅⋅⋅donor distance in **1** is suitable as a chelating ligand to coordinate CuX (X=Cl, Br, I, C_6_F_5_). The distance would undergo adaptive changes in the coordination reaction, especially considering the change of ligand conformation due to the free α‐bond rotation in the solution.

The 2 : 1 reaction of L′Ge with *p*‐diphenol smoothly afforded 1,4‐C_6_H_4_(OGeL)_2_ (**2**, Scheme 2). The melting point of **2** (276.3–278.8 °C) is even slightly higher than that of its meta isomer **1**. The ^1^H NMR of **2** presented a whole set of characteristic resonances for the *β*‐diketiminate ligands, as well as a singlet at *δ* 6.04 ppm denoting the protons on the phenyl bridge with the correct integration. Compound **2** crystallizes in the monoclinic *P*2_1_/*c* space group. The two [LGe] moieties are centrosymmetric with respect to the central phenyl ring (Figure 1b). The two Ge atoms in **2** are arranged in the most opposite positions, seemingly giving the farthest Ge⋅⋅⋅Ge distance (7.937 Å, Figure S1) in such [LGe] systems fused by −OC_6_H_4_O− bridge.

### The reaction of L′Ge with a diol

**Scheme 3:**
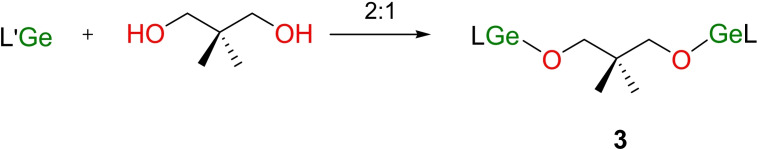
The reaction of L′Ge with neopentyl glycol.

The diphenol type bridge is basically a short and rigid linker. In order to expand the scope of the study, we will consider the introduction of longer or more flexible linkers. For this reason, neopentyl glycol was used as the bridging precursor to react with L′Ge in a ratio of 1 : 2 in toluene solution (Scheme 3) to afford digermylene Me_2_C(CH_2_OGeL)_2_ (**3**).

**Figure 2:**
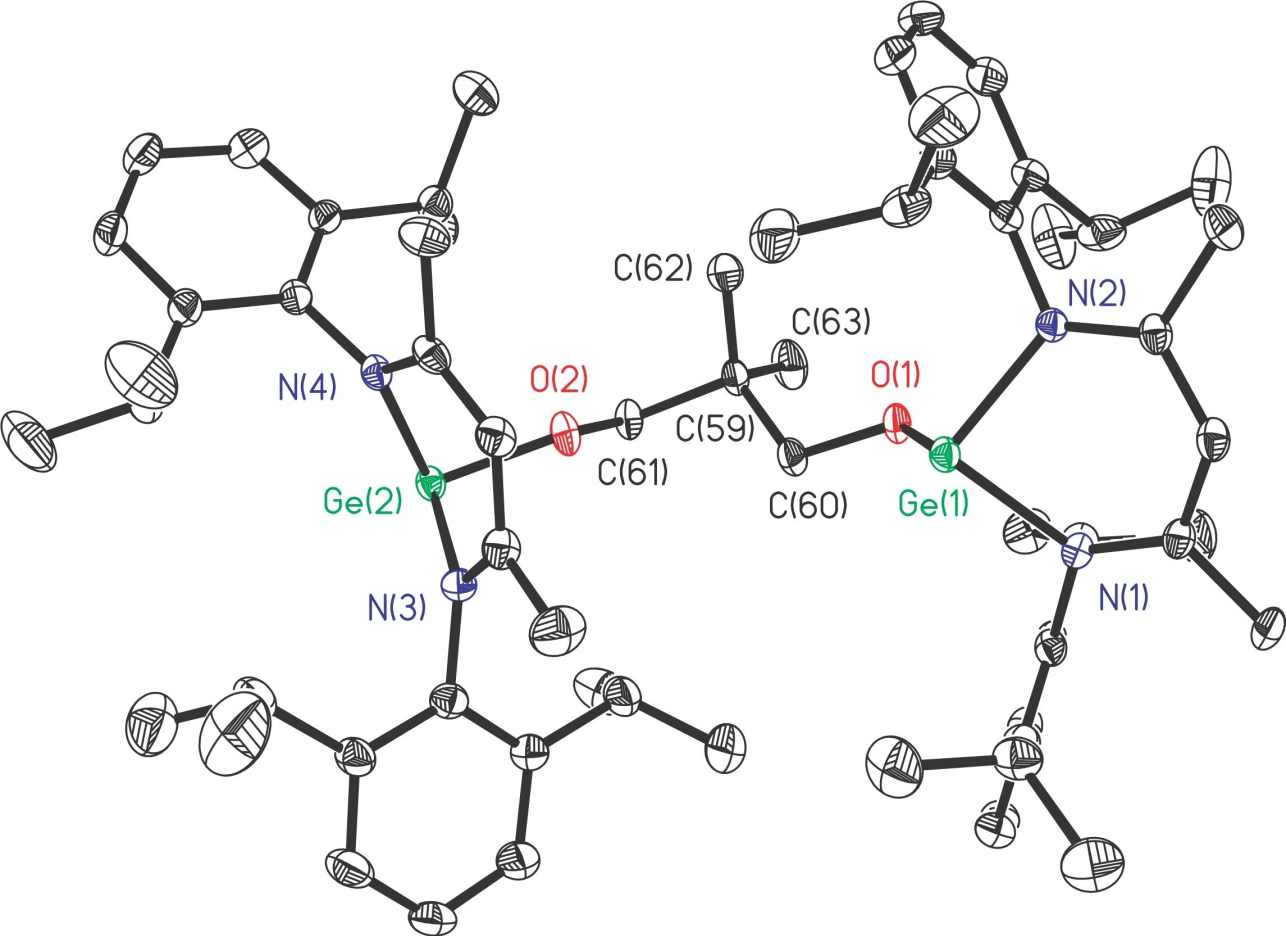
The molecular structure of **3** with the anisotropic displacement parameters is depicted at the 30% probability level. The H atoms are omitted for clarity. Selected bond lengths (Å) and angles (deg): Ge(1)−O(1) 1.818(3), Ge(2)−O(2) 1.839(3); Ge(1)−O(1)−C(60) 116.3(2), Ge(2)−O(2)−C(60) 124.8(2).

The melting point of **3** was found to be dramatically as low as 154.6–156.8 °C, possibly in response to the use of a flexible carbon chain linker.^[25]^ The ^1^H NMR spectrum of **3** features with two singlets at *δ* 2.90 and *δ* 0.14 ppm, representing the proton resonances of methylene (−OC*H*_2_)_2_C and methyl ‐C*M*e_2_ groups, respectively, on the bridging chain. Compound **3** crystallizes in the triclinic space group *P*‐1. In the molecular structure of **3** (Figure 2), the Ge−O bond lengths (1.818(3), 1.839(3) Å) are shorter than those of **1** (1.884(2), 1.849(2) Å) and **2** (1.8494(17) Å), while comparable to that in LGeO*t*Bu (1.827(2) Å]).^[26]^ When comparing with **1** that connects two [LGe] moieties also using −OC_3_O− fragment with the same amount of atoms, the Ge⋅⋅⋅Ge distance (6.770 Å, Figure S1) of **3** is much longer, benefiting from a more stretchable saturated carbon chain. Another interesting observation is that the Ge(2)‐tipped C_2_OGe triangular pyramid points towards the outer sphere of the molecule, in sharp contrast to the situations in **1** and **2**, where the C_2_OGe pyramid pairs point to the inner sphere of the molecule and even roughly point to each other (Figure S3). This outer direction of the Ge donor vector may be useful for the intermolecular assembly in complexation, just like the sulfur ligands did.^[23]^

### The reaction of L′Ge with diamines

**Scheme 4:**
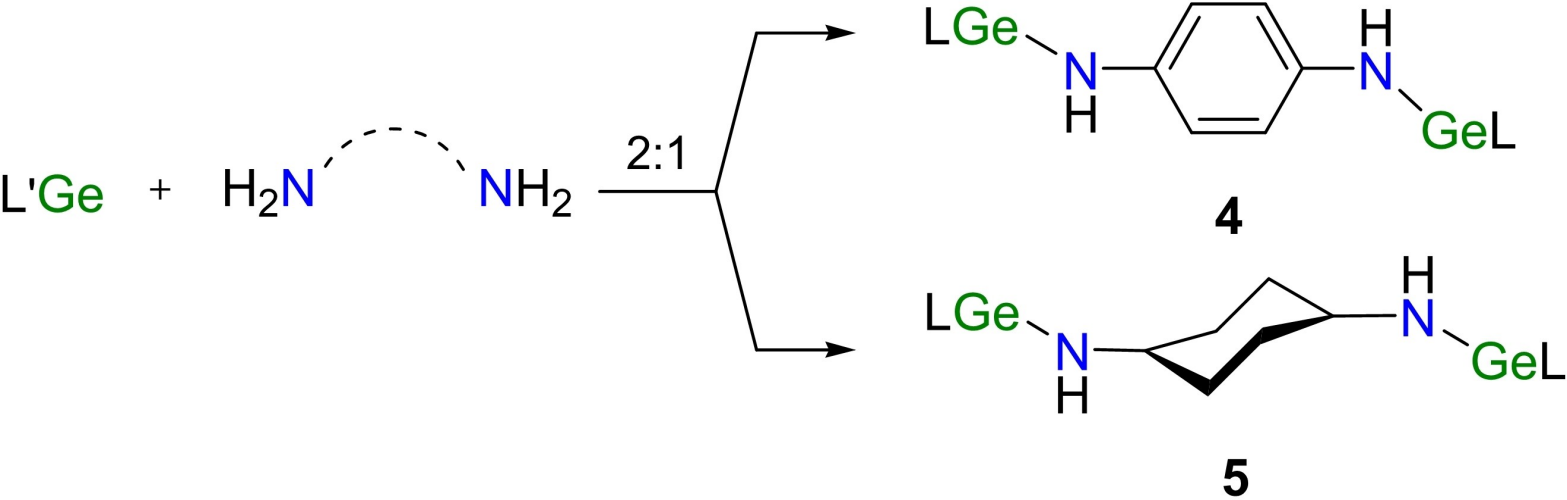
The reactions of L′Ge with *p*‐phenylenediamine and *trans*‐*p*‐cyclohexanediamine.

By widening the reaction of N−H breakage and nucleophilic addition to L′Ge for the synthesis of mononuclear germylene,^[16]^ diamines were employed in the hope of the preparation of new digermylenes. The reaction of L′Ge with *o*‐phenylenediamine also gave free ligand LH, due to the bidentate feature of the latter, just like *o*‐diphenol mentioned above. Treatment of *p*‐phenylenediamine with L′Ge in a 1 : 2 ratio in toluene formed the reddish‐brown solids of 1,4‐C_6_H_4_[N(H)GeL]_2_ (**4**) in a moderate yield (48%) (Scheme 4).

**Figure 3:**
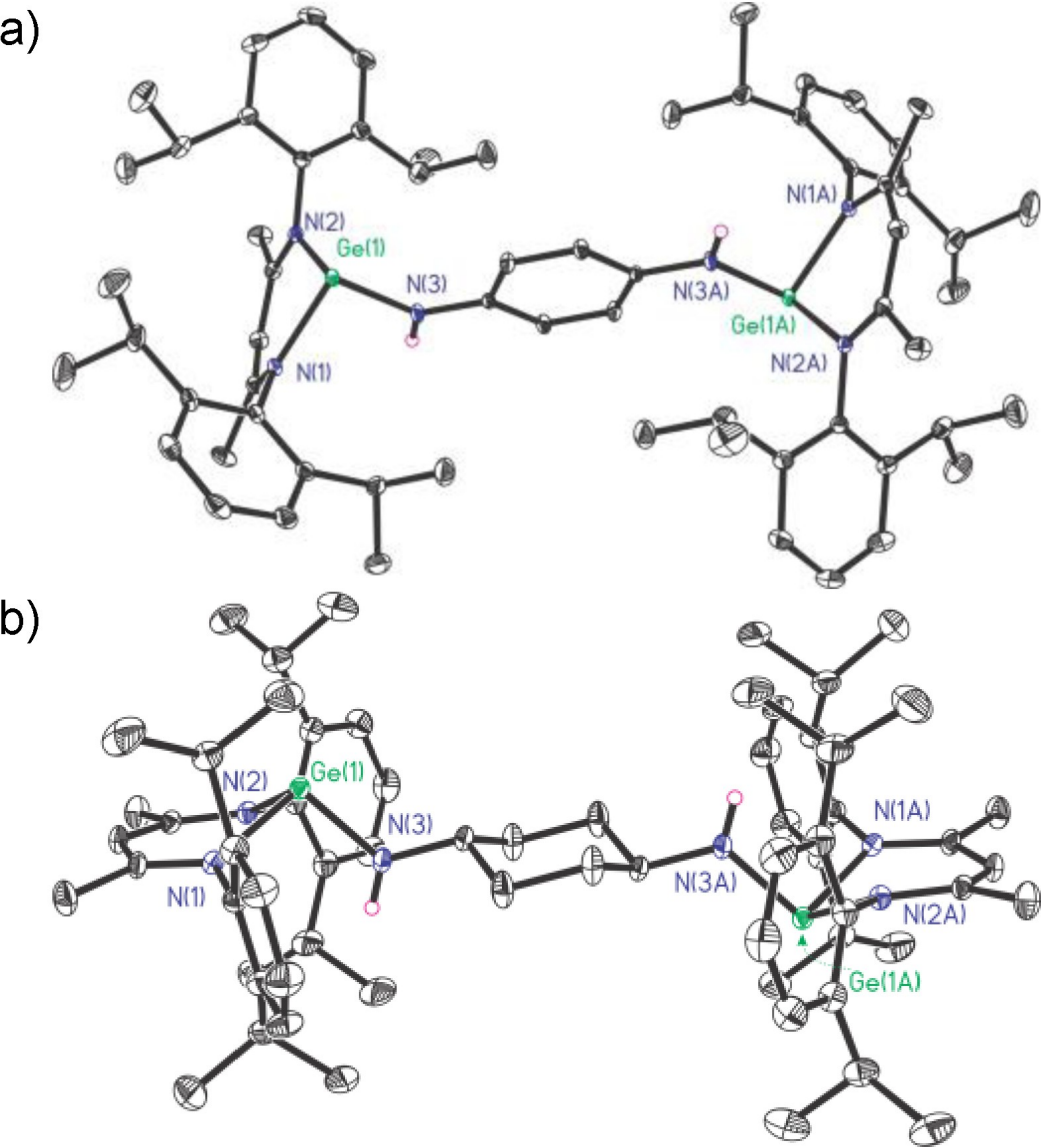
The molecular structures of **4** (a) and **5** (b) with the anisotropic displacement parameters are depicted at the 30% probability level. The H atoms except for NH groups are omitted for clarity. Selected bond lengths (Å) and angles (deg): a) for **4**, Ge(1)−N(3) 1.8823(15); Ge(1)−N(3)−C(30) 127.22(12); b) for **5**, Ge(1)−N(3) 1.839(3); Ge(1)−N(3)−C(30) 121.7(3).

In the ^1^H NMR spectrum of **4**, the singlet at *δ* 4.34 ppm is responsible for the protons of bridging amino groups. The melting range of **4** (290.3–293.6 °C) is almost 15 °C higher than that of its O‐bridged analogue **2**. The saturated solution of **4** in toluene was stored at −20 °C for 5 days to crop red block crystals. Compound **4** crystallizes in the triclinic *P*‐1 space group with an inversion center. As shown in Figure 3, when compared with **2**, the C_3_N_2_ quasi planes of *β*‐diketiminate ligands in **4** are almost vertical to the bridging phenyl plane (84.26°, Figure S1). The Ge⋅⋅⋅Ge separation of **4** (8.481 Å) is slightly longer than that of **2** (7.937 Å), due to the longer N(3)−C and N(3)−Ge bond lengths as well as wider Ge−N−C angle. Both Ge‐tipped pyramids point to the outer sphere of the molecule.

For comparison, the *trans*‐*p*‐cyclohexanediamine with a more flexible framework was used for the synthesis of digermylene 1,4‐C_6_H_10_[N(H)GeL]_2_ (**5**) (Scheme 4). The melting range of **5** (285.6–287.1 °C) is slightly lower than that of **4**. In the ^1^H NMR spectrum of **5**, the resonances appearing at *δ* 0.64–1.54 ppm are assignable to the methine and methylene protons of the bridging cyclohexyl group. The saturated *n*‐hexane solution of **5** at room temperature for 4 days produced red‐brown bulk crystals suitable for X‐ray diffraction analysis. Compound **5** crystallizes in the monoclinic space group *P*2_1_/*c* (Figure 3), bearing an inversion center. The C_3_N_2_ quasi‐planes of the *β*‐diketiminate ligands almost overlap with the calculated cyclohexyl plane. Typically, the cyclohexyl ring adopts a chair conformation. As expected, the Ge⋅⋅⋅Ge distance (8.714 Å, Figure S1) is even longer than that of **4** (8.481 Å).

### The reaction of L′Ge with *o*‐/*m‐*phthalic acids

**Scheme 5:**
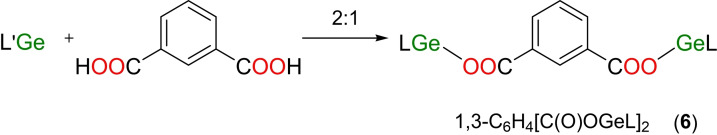
The reaction of L′Ge with *m*‐phthalic acid.

In view of the smooth reaction, L′Ge with PhC(O)OH to generate mononuclear germylene PhC(O)OGeL,^[17]^ the 2 : 1 reactions of L′Ge with *m*‐/*p*‐phthalic acids were carried out for the formation of extended digermylene derivatives (Scheme 5).

**Figure 4:**
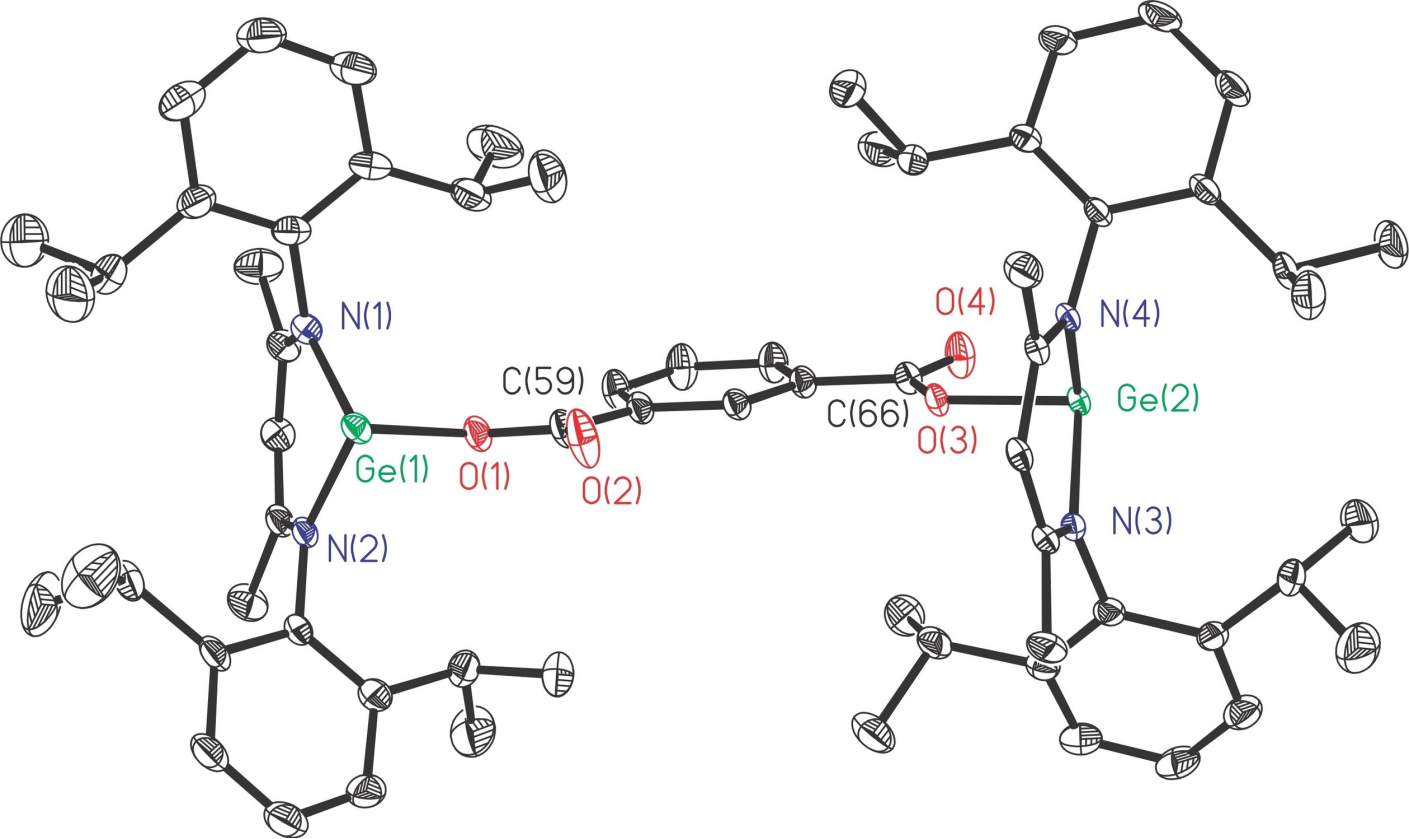
The molecular structure of **6** with the anisotropic displacement parameters is depicted at the 30% probability level. The H atoms are omitted for clarity. Selected bond lengths (Å) and angles (deg): Ge(1)−O(1) 1.925(4), Ge(2)−O(3) 1.942(3); Ge(1)−O(1)−C(59) 122.0 (4), Ge(2)−O(3)−C(66) 115.8(3).

Treatment of L′Ge with *m*‐phthalic acid smoothly afforded pale yellow powders of 1,3‐C_6_H_4_[C(O)OGeL]_2_ (**6**, Scheme 5) in moderate yield (68%). The melting range of **6** (254.4–256.9 °C) is much higher than that of PhC(O)OGeL (167 °C),^[17]^ but it is almost 17 °C lower than that of 1,3‐C_6_H_4_(OGeL)_2_ (**1**). The ^1^H NMR spectrum of **6** exhibits a similar resonance pattern to **1**, while the ^13^C NMR spectrum of **6** confirms the presence of *C*=O resonance at *δ* 169.31 ppm. The molecular structure of **6** is depicted in Figure 4.

Compound **6** crystallizes in the monoclinic *P*2_1_/*n* space group. The two [LGeOOC] moieties are *trans*‐arranged in a way just like that in 1,4‐C_6_H_4_[N(H)GeL]_2_ (**4**). The Ge⋅⋅⋅Ge separation of **6** is further increased to 9.785 Å (Figure S1), much longer than those of **1**–**5**. Another feature of the solid structure of **6** is that the C_3_N_2_Ge rings are largely flat, where the distances of bow Ge atoms deviating from the C_2_N_2_ quasi‐planes are only 0.286 and 0.351 Å (Figure S2).

The product of L′Ge and *p*‐phthalic acid was less soluble in common organic solvents, as precluded further characterization. In contrast, the reaction of L′Ge with *p*‐tert‐butylbenzoic acid proceeded smoothly to give the mononuclear product *p*‐*t*Bu‐C_6_H_4_C(O)OGeL (**7**, Figure S4). One counterintuitive observation in **1**–**6** is that the *γ*‐C and Ge atoms are roughly located on the same plane (Figure S3), regardless of the variation of linker groups.

### Oxidative addition of 1,3‐C_6_H_4_(OGeL)_2_ (1) with sulfur and selenium

**Scheme 6:**
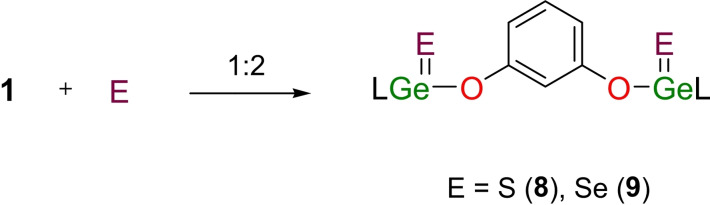
The reactions of 1,3‐C_6_H_4_(OGeL)_2_ (**1**) with sulfur and selenium.

The reactivity of the representative digermylene 1,3‐C_6_H_4_(OGeL)_2_ (**1**) with sulfur and selenium was examined (Scheme 6). The oxidative additions proceeded straightforwardly to produce the products 1,3‐C_6_H_4_[OGe(S)L]_2_ (**8**) and 1,3‐C_6_H_4_[OGe(Se)L]_2_ (**9**), with the ^1^H NMR patterns similar to that of 1,3‐C_6_H_4_(OGeL)_2_ (**1**).

**Figure 5:**
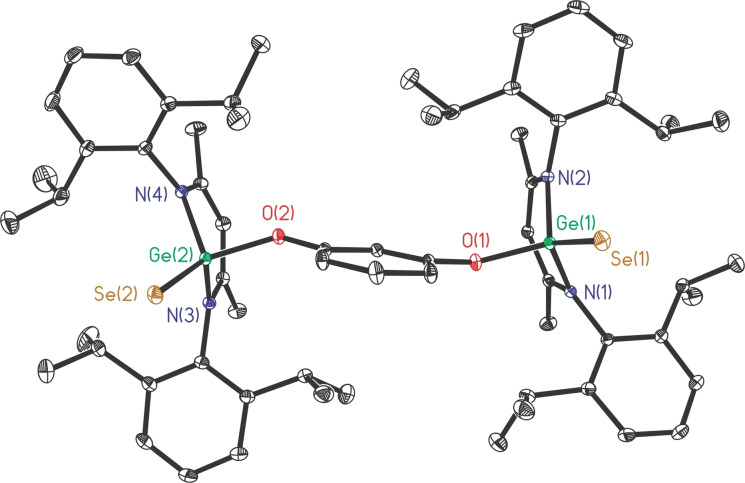
The molecular structure of **9** with the anisotropic displacement parameters is depicted at the 30% probability level. The H atoms are omitted for clarity. Selected bond lengths (Å) and angles (deg): Ge(1)−Se(1) 2.1779(3), Ge(2)−Se(2) 2.1802(3), Ge(1)−O(1) 1.7960(14), Ge(2)−O(2) 1.7903(14); Ge(1)−O(1)−C(60) 133.60(13), Ge(2)−O(2)−C(64) 133.39(13).

The melting points of the obtained Ge(IV) products are nearly 20 °C (**8**) and 50 °C (**9**) higher than that of the parent digermylene **1**. The toluene solutions of **8** and **9**, respectively, were stored at −20 °C for 2 days to result in each being a crop of colorless block crystals. Compound **8** crystallizes in the monoclinic *C*2/*c* space group with unit cell parameters *a*=20.3204(17) Å, *b*=16.3836(13) Å, *c*=48.118(4) Å; *β*=97.378(5)°; *V*=15886.9 Å^3^. However, its crystal data was not complete due to the pulverization during several measurements. In contrast, crystals of compound **9** are robust and crystallize in the triclinic *P*‐1 space group. As shown in Figure 5, each Ge(IV) atom is bonded by two nitrogen, one oxygen, and one selenium atom.

The two [LGe(Se)O] moieties are located in a *cis* arrangement. The Ge−Se bond lengths (2.1779(3), 2.1802(3) Å) are slightly shorter than that of mononuclear LGe(Se)Me (2.199(1) Å).^[27]^ The Ge−O bond lengths (1.7960(14), 1.7903(14) Å) become shorter than those in parent **1** (1.884(2), 1.849(2) Å), as a result of the increase in oxidation state. In addition, Ge−O−C bond angles in **9** (133.60(13), 133.39(13)°) are much wider than those in **1**, helping to increase the Ge⋅⋅⋅Ge distance (8.064 Å, Figure S5) of **9**, which is noticeably longer than that of **1** (5.668 Å).

### Complexation of 1,3‐C_6_H_4_(OGeL)_2_ (1) with CuX

**Scheme 7:**
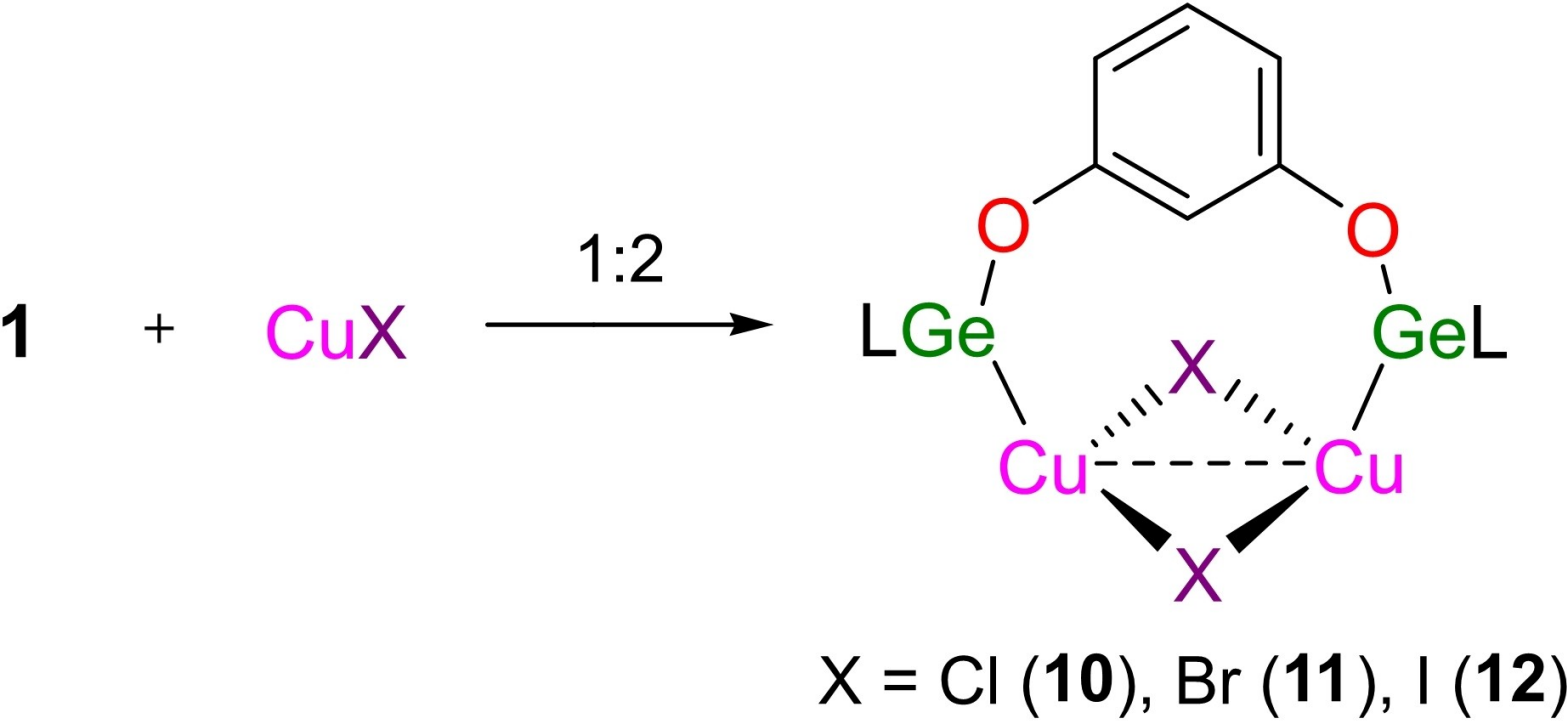
The reaction of 1,3‐C_6_H_4_(OGeL)_2_ (**1**) with CuX (X=Cl, Br, I).

Treatment of 1,3‐C_6_H_4_(OGeL)_2_ (**1**) with CuX (X=Cl, Br, I) in a 1 : 2 ratio generated the corresponding donor‐acceptor complexes (CuX)_2_[1,3‐C_6_H_4_(OGeL)_2_] (X=Cl (**10**), Br (**11**), I (**12**)) (Scheme 7).

**Figure 6:**
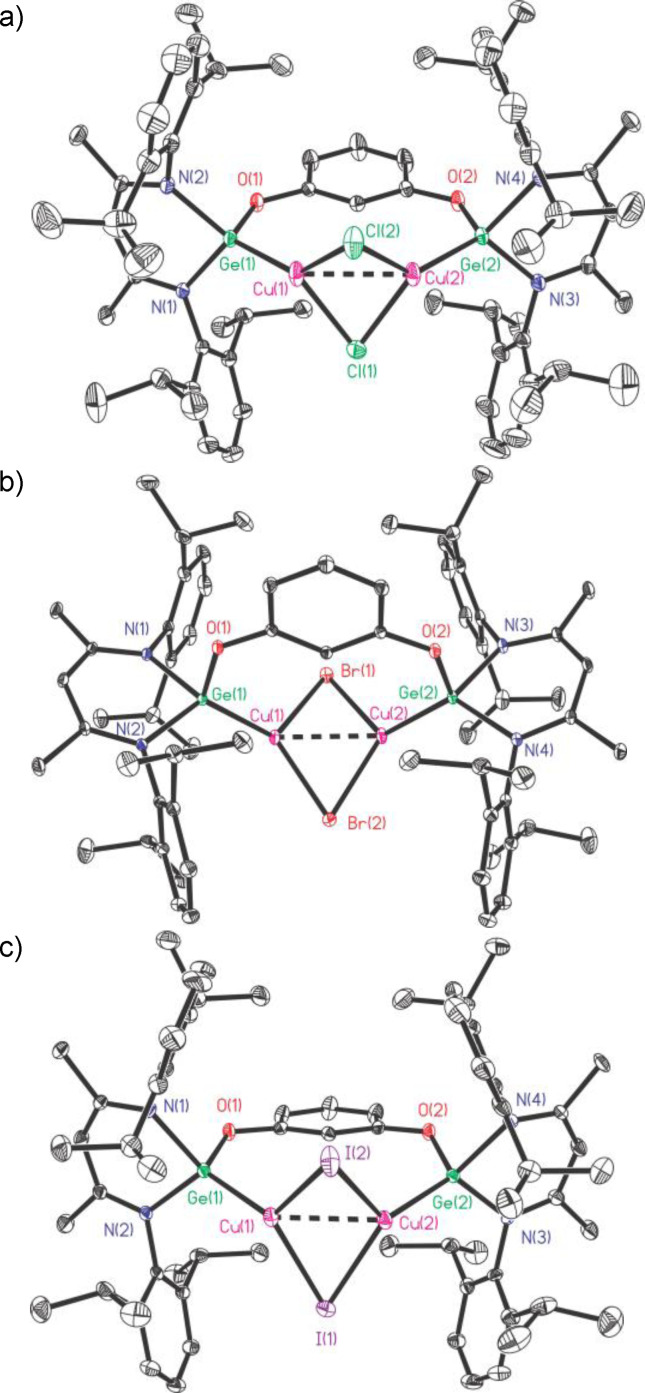
The molecular structures of **10** (a), **11** (b) and **12** (c) with the anisotropic displacement parameters are depicted at the 30% probability level. The H atoms are omitted for clarity. Selected bond lengths (Å) and angles (deg): a) for **10**, Ge(1)−Cu(1) 2.2567(6), Ge(2)−Cu(2) 2.2550(6), Cu(1)−Cu(2) 2.6196(6); Cu(1)−Cl(1)−Cu(2) 68.18(4), Cu(1)−Cl(2)−Cu(2) 71.09(4); b) for **11**, Ge(1)−Cu(1) 2.2677(7), Ge(2)−Cu(2) 2.2643(7), Cu(1)−Cu(2) 2.5852(8); Cu(1)−Br(1)−Cu(2) 63.79(2), Cu(1)−Br(2)−Cu(2) 65.78(2); c) for **12**, Ge(1)−Cu(1) 2.2969(17), Ge(2)−Cu(2) 2.2827(16), Cu(1)−Cu(2) 2.5916(17); Cu(1)−I(1)−Cu(2) 60.66(4), Cu(1)−I(2)−Cu(2) 60.50(4).

The molecular structures of **10**–**12** are shown in Figure 6. They are fundamentally isostructural and share a highly similar structural skeleton (Figure S6), where the halogen atoms are arranged in bridging positions. The common feature of them is that the digermylene **1** therein exhibits chelating properties with two Ge(II) donors to embrace the (CuX)_2_ core, in which each Cu atom is bonded to one Ge and two bridging halogen atoms. The (CuX)_2_ fragments in the molecules of **10**–**12** are significantly twisted as a down‐folded quadrangle, with dihedral angles between Cu_2_X planes 42.35° (**10**), 38.90° (**11**), and 36.98° (**12**), respectively (Figure S7). This observation is totally different from the rhombic plane conformation in (*μ* ^2^‐Cu_2_I_2_)[LGeO*t*Bu]_2_ completed by two discrete mononuclear germylenes.^[20b]^ A comparable example to our work is the germylene oxide supported (CuI⋅pyridine)_2_ bearing the tetracoordinate Cu centers,^[5c]^ with a smaller dihedral angle between Cu_2_I planes (25.61°). From **10** to **12**, both Ge−Cu and Ge−X bond lengths increased with the increase of the atomic number of X, accompanied by a progressive decrease of the Cu−X−Cu angles (Table S1).

One surprising observation is that among **10**–**12** the shortest Cu−Cu bond length (2.5852(8) Å) and Ge⋅⋅⋅Ge distances (5.846 Å) appear in the bromide derivative (**11**), probably due to its Ge−X bond length and Cu−X−Cu bond angle both happen to be relatively small. Nevertheless, all three Cu−Cu bond lengths (2.6196(6), 2.5852(8), 2.5916(17) Å) are shorter when compared with that in [{(*i*Bu)_2_ATIGe}_2_O(Cu_2_I_2_)(C_5_H_5_N)_2_] (2.836 Å).^[5c]^

Furthermore, 1,3‐C_6_H_4_(OGeL)_2_ (**1**) was treated with one equivalent of (CuC_6_F_5_)_4_, producing a partially hydrolyzed product [(F_5_C_6_Cu)_2_OCu_2_][1,3‐C_6_H_4_(OGeL)_2_] (**13**, Figure S8), due to the high moisture sensitivity of this reaction.

### Further exploration of trigermylene

We have shown the feasibility of synthesizing digermylenes using L′Ge with species containing double OH, NH, or C(O)OH functionalities. Along these lines, germylenes with trinuclear or more should also be relatively easy to obtain. Here, we report the preparation of trigermylene using L′Ge with phloroglucinol in a 3 : 1 ratio. As expected, trigermylene 1,3,5‐C_6_H_3_(OGeL)_3_ (**14**) was readily obtained and structurally characterized (Figures S9, S10). The reactivity studies of **14** and the preparation of exciting multinuclear germylenes are in progress.

Moreover, although the digermylene products in this paper are relatively predictable (almost exclusively 1,4‐adducts), at a broader level the study of the reaction pathways of L′Ge with protic and hydridic E−H bonds of HER would still be an issue worthy of further investigation.

## Conclusion

In conclusion, we have demonstrated the feasibility of utilizing L′Ge with diphenols, diol, diamines, and dicarboxylic acids (HE)_2_R (E=O, N, C(O)O) to synthesize the *β*‐diketiminate ligand supported digermylenes R(EGeL)_2_ (L=CH[C(Me)NAr]_2_, Ar=2,6‐*i*Pr_2_C_6_H_3_) (**1**–**6**) by H−E bond cleavage and nucleophilic addition reaction. X‐ray single‐crystal structure analysis revealed that the Ge⋅⋅⋅Ge separations in digermylenes **1**–**6** were affected by the rigidity/flexibility and size of the linker R. The representative digermylene 1,3‐C_6_H_4_(OGeL)_2_ (**1**) exhibited good reactivity with sulfur and selenium, as well as CuX (X=Cl, Br, I, C_6_F_5_) to form donor‐acceptor complexes, where the two Ge donors cooperate to chelate the (CuX)_2_ core. This strategy is also useful in synthesizing multinuclear germylenes and promoting the advance in the assembly and application of Ge→M complexes.

## Experimental Section

### General procedures

All manipulations were carried out in a standard Schlenk technology in a dry argon atmosphere or in the glovebox with oxygen content less than 0.1 ppm. Solvents needed for synthesis in the laboratory were pre‐dried with the molecular sieve 4 Å for 3 days, then added with appropriate Na/K alloy and benzophenone, heated, and refluxed to make the solvents dark blue or purple. After evaporation, the solvents were stored in anhydrous and oxygen‐free solvent bottles in the argon atmosphere for reserve. Compound L′Ge was prepared based on the literature procedure.^[17]^ Other reagents involved in this article were purchased from Aladdin without special instructions.

The melting point of obtained compounds was measured on a digital display micro melting point meter (X‐4B^+^). Infrared spectra were recorded by using KBr pellets with a NEXUS670 (Thermo Fisher Scientific) FT‐IR spectrometer. The ^1^H and ^13^C NMR spectra were measured on a Bruker Advance II 400 MHz NMR instrument at ambient temperature. Elemental analyses for carbon, hydrogen, and nitrogen were performed with a Thermo Quest Italia SPA EA1110 instrument.

### Digermylenes

***1,3‐C***_***6***_ ***H***_***4***_ ***(OGeL)***_***2***_ (**1**). A solution of *m*‐diphenol (0.111 g, 1 mmol) in ether (10 mL) was added to the stirred solution of L′Ge (0.978 g, 2 mmol) in toluene (40 mL) at −78 °C. The solution slowly turns orange. After rising to room temperature, the reaction continued at room temperature for 12 hours, and the volatile substances were dried to obtain yellowish solids. Yield: 0.980 g (0.9 mmol, 90%). M.p.: 271.3–273.8 °C. ^1^H NMR (400 MHz, C_6_D_6_, 298 K, ppm): *δ* 6.96 (m, 12H, Ar‐*H*), 6.54 (s, 1H, Ph‐*H*), 5.76 (s, 2H, Ph‐*H*), 5.40 (s, H, Ph‐*H*), 4.68 (s, 2H, *γ*‐C*H*), 3.48 (sept, *J*=13.4, 6.6 Hz, 4H, C*H*Me_2_), 3.10 (sept, *J*=13.6, 6.8 Hz, 4H, C*H*Me_2_), 1.39 (s, 12H, C*Me*), 1.13 (d, *J*=6.7 Hz, 12H, CH*Me*_2_), 0.99 (d, *J*=6.8 Hz, 12H, CH*Me*_2_), 0.93 (d, *J*=6.8 Hz, 24H, CH*Me*_2_). ^13^C NMR (101 MHz, C_6_D_6_, 298 K, ppm): *δ* 162.57, 161.48, 145.53, 142.61, 139.02 (Ar‐*C*), 128.03 (Ph‐*C*), 123.89, 123.05 (Ar‐*C*), 110.52, 109.85 (Ph‐*C*), 96.53 (*γ*‐*C*), 30.76, 19.34, 27.95, 27.33 (*C*HMe_2_), 24.68 (*C*HMe_2_), 24.33, 23.66, 23.55 (CH*Me*_2_), 22.00 (C*Me*). FT‐IR (cm^−1^): ν 3448.34 (w), 3054.84 (w), 2966.13 (s), 2921.77 (m), 2869.71 (w), 1618.63 (vw), 1560.21 (s), 1475.35 (w), 1380.85 (w), 1319.36 (m), 1174.49 (m), 1137.35 (w), 1103.14 (w), 1018.28 (w), 971.99 (m), 856.28 (vw), 790.49 (s), 755.99 (w), 694.28 (vw), 619.07 (w), 586.28 (w), 526.49 (vw). Anal. Calcd. for C_64_H_86_Ge_2_N_4_O_2_ (1088.68): C, 70.61; H, 7.96; N, 5.15%; found C, 70.37; H, 7.83; N, 4.96%. A pale‐yellow crystal suitable for single‐crystal X‐ray diffraction was obtained by dissolving the solid compound **1** in toluene solution and concentrating it to 3 mL, then dropping 2 mL of *n*‐hexane solution and storing the mixture solution in a refrigerator at −20 °C for 4 days.

***1,4‐C***_***6***_ ***H***_***4***_ ***(OGeL)***_***2***_ (**2**). The *p*‐diphenol (0.111 g, 1 mmol) and L′Ge (0.978 g, 2 mmol) were used for the synthesis of **2**. Yield: 0.936 g (0.86 mmol, 86%). M.p.: 276.3–278.8 °C. ^1^H NMR (400 MHz, C_6_D_6_, 298 K, ppm): *δ* 6.93 (s, 12H, Ar‐*H*), 6.04 (s, 4H, Ph‐*H*), 4.71 (s, 2H, *γ*‐C*H*), 3.52 (sept, *J*=13.4, 6.6 Hz, 4H, C*H*Me_2_), 3.05 (sept, *J*=13.6, 6.8 Hz, 4H, C*H*Me_2_), 1.36 (s, 12H, C*Me*), 1.05 (d, *J*=6.7 Hz, 12H, CH*Me*_2_), 0.96 (d, *J*=6.8 Hz, 12H, CH*Me*_2_), 0.86 (d, *J*=6.8 Hz, 12H, CH*Me*_2_), 0.84 (d, *J*=6.8 Hz, 12H, CH*Me*_2_). ^13^C NMR (101 MHz, C_6_D_6_, 298 K, ppm): *δ* 163.52, 146.43, 143.53, 140.20, 124.67, 123.76, 119.20 (Ar/Ph‐*C*), 97.92 (*γ*‐*C*), 57.37, 28.76, 28.00 (*C*HMe_2_), 25.93, 24.94, 24.31 (CH*Me*_2_), 22.95 (C*Me*). FT‐IR (cm^−1^): ν 3444.41 (w), 2964.23 (w), 2871.63 (w), 1629.63 (w), 1548.64 (w), 1467.64 (w), 1380.85 (w), 1324.92 (w), 1236.21 (w), 1103.14 (w), 1018.28 (w), 969.43 (w), 857.31 (vw), 791.21 (s), 786.85 (w), 759.85 (w), 694.28 (vw), 619.07 (vw), 586.28 (vw), 524.57 (vw). Anal. Calcd. for C_64_H_86_Ge_2_N_4_O_2_ (1088.68): C, 70.61; H, 7.96; N, 5.15%; found C, 70.47; H, 7.86; N, 5.06%. A pale‐yellow crystal suitable for single‐crystal X‐ray diffraction was obtained by storing the saturated toluene solution of compound **2** in a refrigerator at −20 °C for 7 days.

***Me***_***2***_ ***C(CH***_***2***_ ***OGeL)***_***2***_ (**3**). The neopentyl glycol (0.104 g, 1 mmol) and L′Ge (0.978 g, 2 mmol) were used for the synthesis of **3**. Yield: 0.866 g (0.8 mmol, 80%). M.p.: 154.6–156.8 °C. ^1^H NMR (400 MHz, C_6_D_6_, 298 K, ppm): *δ* 6.96–7.00 (m, 12H, Ar‐*H*), 4.55 (s, 2H, *γ*‐C*H*), 3.56 (sept, *J*=13.5, 6.7 Hz, 4H, C*H*Me_2_), 3.09 (sept, *J*=13.5, 6.7 Hz, 4H, C*H*Me_2_), 2.90 (s, 4H, OC*H*_2_), 1.31 (s, 12H, C*Me*), 1.21 (d, *J*=6.7 Hz, 12H, CH*Me*_2_), 1.08 (d, *J*=6.8 Hz, 12H, CH*Me*_2_), 1.08 (d, *J*=6.8 Hz, 12H, CH*Me*_2_), 0.93 (d, *J*=6.8 Hz, 12H, CH*Me*_2_), 0.14 (s, 6H, C*Me*_2_). ^13^C NMR (101 MHz, C_6_D_6_, 298 K, ppm): *δ* 162.57, 144.32, 143.10, 139.27, 125.93, 123.39, 123.22 (Ar‐*C*), 95.90 (*γ*‐*C*), 71.65 (O*C*H_2_), 38.49 (*C*Me_2_), 27.59, 27.26 (*C*HMe_2_), 25.25 (*C*HMe_2_), 24.12, 23.71, 23.50, 22.23 (C*Me*), 21.59, 20.98 (CH*Me*_2_). FT‐IR (cm^−1^): ν 3417.41 (w), 3060.62 (w), 2958.41 (s), 2861.98 (m), 1625.78 (w), 1558.28 (w), 1461.82 (w), 1386.64 (vs), 1317.21 (s), 1259.35 (m), 1078.07 (s), 1020.21 (s), 991.28 (vw), 937.28 (w), 850.50 (w), 794.57 (vw), 754.07 (vw), 673.07 (m), 765.43 (w), 638.35 (vw), 518.78 (vw). Anal. Calcd. for C_63_H_92_Ge_2_N_4_O_2_ (1082.72): C, 69.89; H, 8.57; N, 5.17%; found C, 69.78; H, 8.43; N, 5.01%. Compound **3** was placed in concentrated n‐hexane solvent for 3 days to obtain pale yellow crystals suitable for single‐crystal X‐ray diffraction analysis.

***1,4‐C***_***6***_ ***H***_***4***_ ***[N(H)GeL]***_***2***_ (**4**). The 1,4‐C_6_H_4_(NH)_2_ (0.108 g, 1 mmol) and L′Ge (0.978 g, 2 mmol) were used for the synthesis of **4**. Yield: 0.521 g (0.48 mmol, 48%). M.p.: 290.3–293.6 °C. ^1^H NMR (400 MHz, C_6_D_6_, 298 K, ppm): *δ* 6.74–7.13 (m, 12H, Ar‐*H*), 6.09 (s, 4H, Ph‐*H*), 4.64 (s, 2H, *γ*‐C*H*), 4.34 (br, 2H, N*H*), 3.31 (sept, *J*=13.4, 6.6 Hz, 4H, C*H*Me_2_), 3.20 (sept, *J*=13.6, 6.8 Hz, 4H, C*H*Me_2_), 1.37 (s, 12H, C*Me*), 1.12 (d, *J*=6.7 Hz, 12H, CH*Me*_2_), 1.01 (d, *J*=6.8 Hz, 12H, CH*Me*_2_), 0.91 (d, *J*=6.8 Hz, 12H, CH*Me*_2_), 0.87 (d, *J*=6.8 Hz, 12H, CH*Me*_2_). ^13^C NMR (101 MHz, C_6_D_6_, 298 K, ppm): *δ* 163.46, 146.35, 143.30, 141.38, 124.57, 123.73, 116.21 (Ar/Ph‐*C*), 96.18 (*γ*‐*C*), 28.88, 28.50 (*C*HMe_2_), 26.57, 24.38, 24.03 (CH*Me*_2_), 23.17 (C*Me*). FT‐IR (cm^−1^): ν 3455.98 (w), 3060.63 (vw), 2960.34 (w), 2927.56 (w), 2871.63 (w), 1621.92 (w), 1550.56 (w), 1496.56 (w), 1459.71 (w), 1442.56 (w), 1390.49 (w), 1317.21 (w), 1263.21 (w), 1170.64 (w), 1103.99 (w), 1027.92 (w), 937.49 (vw), 850.49 (vw), 798.42 (w), 755.99 (w), 746.35 (w), 723.31 (vw), 586.71 (w), 518.78 (vw). Anal. Calcd. for C_64_H_88_Ge_2_N_6_ (1086.71): C, 70.74; H, 8.16; N, 7.73%; found C, 70.87; H, 8.09; N, 7.57%.

***1,4‐C***_***6***_ ***H***_***10***_ ***[N(H)GeL]***_***2***_ (**5**). The *trans*‐*p*‐cyclohexanediamine (0.114 g, 1 mmol) and L′Ge (0.978 g, 2 mmol) were used for the synthesis of **5**. Yield: 0.764 g (0.70 mmol, 70%). M.p.: 285.6–287.1 °C. ^1^H NMR (400 MHz, C_6_D_6_, 298 K, ppm): *δ* 7.00–7.07 (m, 12H, Ar‐*H*), 4.64 (s, 2H, *γ*‐C*H*), 3.55 (sept, *J*=13.4, 6.6 Hz, 4H, CH*Me*_2_), 3.38 (sept, *J*=13.6, 6.8 Hz, 4H, CH*Me*_2_), 2.38 (s, 2H, N*H*), 1.54 (s, 2H, C*H*), 1.49 (s, 12H, C*Me*), 1.31 (d, *J*=6.7 Hz, 12H, CH*Me*_2_), 1.26 (d, *J*=6.8 Hz, 12H, CH*Me*_2_), 1.13 (d, *J*=6.8 Hz, 12H, CH*Me*_2_), 1.12 (d, *J*=6.8 Hz, 12H, CH*Me*_2_), 0.96 (m, 4H, C*H*_2_), 0.64 (m, 4H, C*H*_2_). ^13^C NMR (101 MHz, C_6_D_6_, 298 K, ppm): *δ* 163.50, 143.75, 142.91, 139.48, 125.31, 123.48, 122.74 (Ar‐*C*), 94.32 (*γ*‐*C*), 51.92 (*C*H), 38.23 (*C*H_2_), 27.54 (*C*HMe_2_), 25.55, 23.92 (CH*Me*_2_), 23.61, 23.44 (C*Me*), 22.95 (*C*HMe_2_), 22.27 (CH*Me*_2_), 21.91. FT‐IR (cm^−1^): ν 3446.34 (w), 3058.70 (vw), 2960.34 (w), 2925.63 (w), 2861.99 (w), 1569.85 (m), 1523.56 (w), 1465.71 (w), 1442.56 (w), 1384.71 (w), 1315.28 (w), 1253.56 (w), 1170.64 (w), 1106.99 (w), 1054.92 (w), 1022.14 (w), 958.49 (vw), 850.49 (vw), 798.42 (w), 746.35 (w), 723.31 (vw), 628.71 (w), 514.93 (vw). Anal. Calcd. for C_64_H_94_Ge_2_N_6_ (1092.76): C, 70.35; H, 8.67; N, 7.69%; found C, 70.37; H, 8.83; N, 7.51%. Red bulk crystals for single‐crystal X‐ray diffraction were obtained from a saturated *n*‐hexane solution at room temperature after 7 days.

***1,3‐C***_***6***_ ***H***_***4***_ ***[C(O)OGeL]***_***2***_ (**6**). The *m*‐phthalic acid (0.166 g, 1 mmol) and L′Ge (0.978 g, 2 mmol) were used for the synthesis of **6**. Yield: 0.778 g (0.68 mmol, 68%). M.p.: 254.4–256.9 °C. ^1^H NMR (400 MHz, C_6_D_6_, 298 K, ppm): *δ* 9.02 (s, 1H, Ph‐*H*), 8.25 (m, 2H, Ph‐*H*), 7.00 (s, 1H, Ph‐*H*), 6.83–6.98 (m, 12H, Ar‐*H*), 4.92 (s, 2H, *γ*‐C*H*), 3.45 (sept, *J*=6.6 Hz, 4H, C*H*Me_2_), 2.96 (sept, *J*=13.6, 6.8 Hz, 4H, C*H*Me_2_), 1.40 (s, 12H, C*Me*), 0.95–0.99 (m, 36H, CH*Me*_2_), 0.84 (d, *J*=6.8 Hz, 12H, CH*Me*_2_). ^13^C NMR (101 MHz, C_6_D_6_, 298 K, ppm): *δ* 168.23 (Ar‐*C*), 163.91 (*C*=O), 145.47, 142.47, 139.40, 135.05, 131.01, 124.04, 122.99 (Ar/Ph‐*C*), 98.90 (*γ*‐*C*), 30.76, 28.21, 27.15 (*C*HMe_2_), 25.43, 23.53, 23.40, 23.14, 22.95 (CH*Me*_2_), 22.27 (C*Me*). FT‐IR (cm^−1^): ν 3425.19 (w), 3130.98 (w), 3065.41 (vw), 2967.16 (w), 2859.23. (w), 1716.63 (w), 1646.71 (w), 1527.99 (s), 1465.71 (w), 1442.56 (w), 1376.64 (w), 1319.14 (w), 1257.42 (w), 1173.42 (w), 1096.35 (w), 1015.34 (w), 932.41 (w), 857.28 (w), 796.35 (vw), 757.92 (s), 732.64 (w), 696.14 (w), 645.99 (vw), 587.21 (w), 570.82 (vw), 525.51 (vw). Anal. Calcd. for C_66_H_86_Ge_2_N_4_O_4_ (1144.70): C, 69.25; H, 7.57; N, 4.89%; found C, 69.32; H, 7.63; N, 4.96%. The colorless bulk crystals of **6** precipitated on the surface of the bottle during slow volatilization of THF.

### Oxidative addition products of 1 with sulfur and selenium

***1,3‐C***_***6***_ ***H***_***4***_ ***[OGe(S)L]***_***2***_ (**8**). A solution of 1,3‐C_6_H_4_(OGeL)_2_ (0.545 g, 0.5 mmol) and sulfur (0.032 g, 1 mmol) in toluene (40 mL) was stirred at 85 °C for 12 hours. Yield: 0.530 g (0.46 mmol, 91%). M.p.: 290.2–291.9 °C. ^1^H NMR (400 MHz, C_6_D_6_, 298 K, ppm): δ=7.12 (m, 12H, Ar‐*H*), 6.91 (m, 2H, Ph‐*H*), 6.74 (m, 1H, Ph‐*H*), 6.52 (s, 1H, Ph‐*H*), 4.87 (s, 2H, *γ*‐C*H*), 3.45–3.53 (m, 4H, C*H*Me_2_), 1.55 (s, 12H, C*Me*), 1.51 (br, 12H, CH*Me_2_*), 1.15–1.18 (dd, *J*=6.7 Hz, 24H, CH*Me*_2_), 1.08 (d, *J*=6.8 Hz, 12H, CH*Me*_2_). ^13^C NMR (101 MHz, C_6_D_6_, 298 K, ppm) *δ* 169.65, 158.83, 146.24, 145.21 (Ar‐*C*), 137.65 (Ph‐*C*), 136.92, 128.06, 125.45, 124.83, 124.61 (Ar‐*C*), 113.19, 112.41 (Ph‐*C*), 99.21 (*γ*‐*C*), 29.15, 28.54, 25.71, 25.45, 24.55, 23.80, 21.20 (*C*HMe_2_, CH*Me*_2_, and C*Me*). FT‐IR (cm^−1^): ν 3456.34 (w), 3051.84 (w), 2960.13 (s), 2919.71 (m), 2860.72 (w), 1620.23 (vw), 1483.26 (w), 1386.25 (w), 1316.32 (m), 1178.29 (m), 1140.35 (w), 1104.14 (w), 1018.28 (w), 970.99 (m), 856.28 (vw), 793.49 (s), 752.99 (w), 694.28 (vw), 620.07 (w), 587.28 (w), 552.43 (vw) 526.49 (vw). Anal. Calcd. for C_64_H_86_Ge_2_N_4_O_2_S_2_ (1152.80): C, 66.68; H, 7.52; N, 4.86%; found C, 66.55; H, 7.75; N, 4.75%.

***1,3‐C***_***6***_ ***H***_***4***_ ***[OGe(Se)L]***_***2***_ (**9**). A solution of 1,3‐C_6_H_4_(OGeL)_2_ (0.545 g, 0.5 mmol) and selenium (0.078 g, 1 mmol) in toluene (40 mL) was stirred at 85 °C for 12 hours. Yield: 0.561 g (0.45 mmol, 94%). M.p.: 320.4–321.9 °C. ^1^H NMR (400 MHz, C_6_D_6_, 298 K, ppm): *δ* 6.90–7.13 (m, 12H, Ar‐*H*), 6.73 (m, 2H, Ph‐*H*), 6.49 (m, 1H, Ph‐*H*), 6.35 (s, 1H, Ph‐*H*), 4.67 (s, 2H, *γ*‐C*H*), 3.28–3.34 (m, 4H, C*H*Me_2_), 1.36 (d, *J*=6.7 Hz, 12H, CH*Me*_2_), 1.31 (s, 12H, C*Me*), 0.98–1.00 (dd, *J*=6.8 Hz, 24H, CH*Me*_2_), 0.89 (d, *J*=6.8 Hz, 12H, CH*Me*_2_). ^13^C NMR (101 MHz, C_6_D_6_, 298 K, ppm) *δ* 163.52, 146.43, 143.53, 140.20, 136.34, 124.67, 123.76, 119.20 (Ar/Ph‐*C*), 97.92 (*γ*‐*C*), 28.76, 28.01, 25.93, 24.94, 24.30, 22.95 (*C*HMe_2_, CH*Me*_2_, and C*Me*). FT‐IR (cm^−1^): ν 3456.34 (w), 3051.47 (w), 2960.13 (s), 2860.72 (w), 1620.23 (vw), 1542.71 (vw), 1480.46 (w), 1360.52 (w), 1316.32 (m), 1182.29 (m), 1140.35 (w), 1106.15 (w), 1019.28 (w), 970.99 (m), 856.28 (vw), 794.49 (s), 747.99 (w), 694.28 (vw), 620.07 (w), 587.28 (w), 552.43 (vw) 526.49 (vw). Anal. Calcd. for C_64_H_86_Ge_2_N_4_O_2_Se_2_ (1246.60): C, 61.66; H, 6.95; N, 4.49%; found C, 61.55; H, 6.75; N, 4.35%. The concentrated solution was stored at −20 °C in a freezer for 2 days to get the colorless and transparent massive crystals.

### Coordination products of 1 with CuX

***(CuCl)***_***2***_ ***[1,3‐C***_***6***_ ***H***_***4***_ ***(OGeL)***_***2***_ ***]*** (**10**) The 1,3‐C_6_H_4_(OGeL)_2_ (0.545 g, 0.5 mmol) and CuCl (0.098 g, 1 mmol) were used for the synthesis of **10**. Yield: 0.547 g (0.425 mmol, 85%). M.p.: 337.4–338.9 °C. ^1^H NMR (400 MHz, C_6_D_6_, 298 K, ppm) *δ* 6.93–7.13 (m, 14H, Ar/Ph‐*H*), 6.53 (m, 2H, Ph‐*H*), 4.91 (s, 2H, *γ*‐C*H*), 3.46–3.52 (m, 4H, C*H*Me_2_), 3.11–3.14 (m, 4H, C*H*Me_2_), 1.51 (s, 12H, C*Me*), 1.49 (d, *J*=6.7 Hz, 12H, CH*Me*_2_), 1.23 (d, *J*=6.6 Hz, 12H, CH*Me*_2_), 0.94–0.96 (dd, *J*=20.9, 6.3 Hz, 24H, CH*Me*_2_).^13^C NMR (101 MHz, C_6_D_6_, 298 K, ppm) *δ* 165.91, 160.54, 144.96, 142.29, 137.44, 136.69, 128.13, 127.51, 127.36, 124.49, 123.88, 123.43 (Ar/Ph‐*C*), 110.90 (*γ*‐C), 28.78, 27.05, 26.07, 23.54, 22.57, 20.24 (*C*HMe_2_, CH*Me*_2_, and C*Me*). FT‐IR (cm^−1^): ν 3447.45 (w), 3033.56 (w), 2966.13 (s), 2921.77 (m), 2868.34 (w), 1617.34 (vw), 1560.21 (s), 1475.35 (w), 1380.85 (w), 1319.36 (m), 1174.49 (m), 1137.35 (w), 1102.78 (w), 1018.28 (w), 971.99 (m), 856.28 (vw), 790.49 (s), 758.34 (w), 692.56 (vw), 618.52 (w), 586.28 (w), 527.35 (vw). Anal. Calcd. for C_64_H_86_Ge_2_N_4_O_2_Cu_2_Cl_2_ (1286.67): C, 59.74; H, 6.74; N, 4.35%; found C, 59.87; H, 6.75; N, 4.25%.

***(CuBr)***_***2***_ ***[1,3‐C***_***6***_ ***H***_***4***_ ***(OGeL)***_***2***_ ***]*** (**11**). The 1,3‐C_6_H_4_(OGeL)_2_ (0.545 g, 0.5 mmol) and CuBr (0.143 g, 1 mmol) were used for the synthesis of **11**. Yield: 0.591 g (0.430 mmol, 86%). M.p.: 340.2–341.6 °C. ^1^H NMR (400 MHz, C_6_D_6_, 298 K, ppm) *δ* 7.00–7.09 (m, 14H, Ar/Ph‐*H*), 6.56 (d, *J*=8.0 Hz, 2H, Ph‐*H*), 4.92 (s, 2H, *γ*‐C*H*), 3.45–3.57 (m, 4H, C*H*Me_2_), 3.09–3.23 (m, 4H, C*H*Me_2_), 1.54 (br, 24H, C*Me* and CH*Me*_2_), 1.27 (d, *J*=6.2 Hz, 12H, CH*Me*_2_), 0.93–0.95 (dd, *J*=25.8, 6.0 Hz, 24H, CH*Me*_2_). ^13^C NMR (101 MHz, C_6_D_6_, 298 K, ppm) *δ* 162.57, 161.49, 145.54, 142.61, 139.02, 128.03, 126.44, 123.89, 122.67, 121.37 (Ar/Ph‐*C*), 110.52, 109.85 (Ph‐*C*), 96.53 (*γ*‐*C*), 29.34, 27.95, 27.33, 24.69, 24.33, 23.55, 20.00 (*C*HMe_2_, CH*Me*_2_, and C*Me*). FT‐IR (cm^−1^): ν 3447.35 (w), 3054.84 (w), 2989.24 (s), 2873.68 (m), 2869.71 (w), 1618.63 (vw), 1553.85 (s), 1475.35 (w), 1380.85 (w), 1317.38 (m), 1256.49 (m), 1120.57 (w), 1005.14 (w), 1018.28 (w), 971.99 (m), 856.28 (vw), 790.49 (s), 748.78 (w), 694.28 (vw), 628.47 (w), 594.39 (w), 526.49 (vw). Anal. Calcd. for C_64_H_86_Ge_2_N_4_O_2_Cu_2_Br_2_ (1375.58): C, 55.88; H, 6.30; N, 4.07%; found C, 55.87; H, 6.17; N, 3.97%.

***(CuI)***_***2***_ ***[1,3‐C***_***6***_ ***H***_***4***_ ***(OGeL)***_***2***_ ***]*** (**12**). The 1,3‐C_6_H_4_(OGeL)_2_ (0.545 g, 0.5 mmol) and CuI (0.190 g, 1 mmol) were used for the synthesis of **12**. Yield: 0.566 g (0.385 mmol, 77%). M.p.: 345.2–347.8 °C. ^1^H NMR (400 MHz, C_6_D_6_, 298 K, ppm): *δ* 6.96 (m, 12H, Ar‐*H*), 6.54 (s, 1H, Ph‐*H*), 5.76 (s, 2H, Ph‐*H*), 5.40 (s, H, Ph‐*H*), 4.68 (s, 2H, *γ*‐C*H*), 3.48 (sept, *J*=13.4, 6.6 Hz, 4H, C*H*Me_2_), 3.10 (sept, *J*=13.6, 6.8 Hz, 4H, C*H*Me_2_), 1.39 (s, 12H, C*Me*), 1.13 (d, *J*=6.7 Hz, 12H, CH*Me*_2_), 0.93 (d, *J*=6.8 Hz, 12H, CH*Me*_2_), 0.91 (d, *J*=6.8 Hz, 12H, CH*Me*_2_), 0.80 (d, *J*=6.8 Hz, 12H, CH*Me*_2_). ^13^C NMR (101 MHz, C_6_D_6_, 298 K, ppm) *δ* 165.91, 160.53, 144.96, 142.93, 137.44, 136.69, 128.13, 127.52, 127.37, 124.49, 123.88, 123.43 (Ar/Ph‐*C*), 110.90 (*γ*‐*C*), 28.78, 27.06, 26.07, 23.54, 22.57, 20.24 (*C*HMe_2_, CH*Me*_2_, and C*Me*). FT‐IR (cm^−1^): ν 3448.34 (w), 3054.84 (w), 2966.13 (s), 2921.77 (m), 2869.71 (w), 1618.63 (vw), 1560.21 (s), 1475.35 (w), 1380.85 (w), 1319.36 (m), 1174.49 (m), 1137.35 (w), 1103.14 (w), 1018.28 (w), 971.99 (m), 856.28 (vw), 790.49 (s), 755.99 (w), 694.28 (vw), 619.07 (w), 586.28 (w), 526.49 (vw). Anal. Calcd. for C_64_H_86_Ge_2_N_4_O_2_Cu_2_I_2_ (1469.58): C, 52.31; H, 5.90; N, 3.81%; found C, 52.43; H, 5.88; N, 3.82%.

### X‐ray crystallographic analysis

The crystallographic data were measured on a Bruker SMART APEX II CCD single crystal diffractometer with MoKα radiation (**1**–**7**, **9**, **10**, and **12**–**14**) and an XtaLAB Synergy, Dualflex, HyPix diffractometer from Rigaku with CuKα radiation (**11**). The data were refined by SHELXL,^[28]^ and all non‐proton coordinate values and anisotropic temperature factors were refined by the full‐matrix least‐squares method. All proton positions in the structure were determined by theoretical calculation

## Conflict of interest

The authors declare no conflict of interest.

1

## Supporting information

As a service to our authors and readers, this journal provides supporting information supplied by the authors. Such materials are peer reviewed and may be re‐organized for online delivery, but are not copy‐edited or typeset. Technical support issues arising from supporting information (other than missing files) should be addressed to the authors.

Supporting Information

## Data Availability

The data that support the findings of this study are available in the supplementary material of this article.
