# Supplementary material for: Synthesis, Characterization, and Reaction of Digermylenes
Source: Chem Asian J. 2022 May 16;17(13):e202200141. doi: 10.1002/asia.202200141 (PMC9400958; doi:10.1002/asia.202200141)
Supplement: Supplementary file 1 — Supporting Information [file ASIA-17-0-s001.pdf]

CHEMISTRY
AN ASIAN JOURNAL

Supporting Information

Synthesis, Characterization, and Reaction of Digermylenes

Shuai-Cong Huo[+], Yao Li[+], De-Xiang Zhang, Qi Zhou, Ying Yang,* and Herbert W. Roesky*© 2022 The Authors. Chemistry - An Asian Journal published by Wiley-VCH GmbH. This is an open access article under the terms of the Creative Commons Attribution License, which permits use, distribution and reproduction in any medium, provided the original work is properly cited.

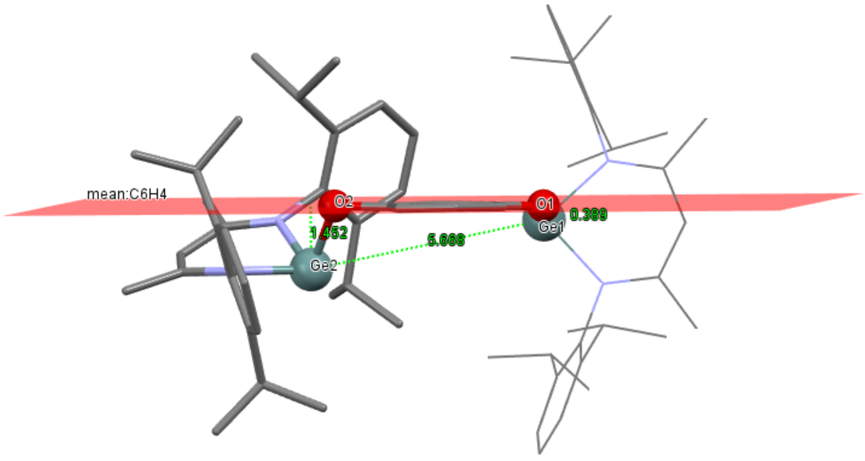

(**1**)

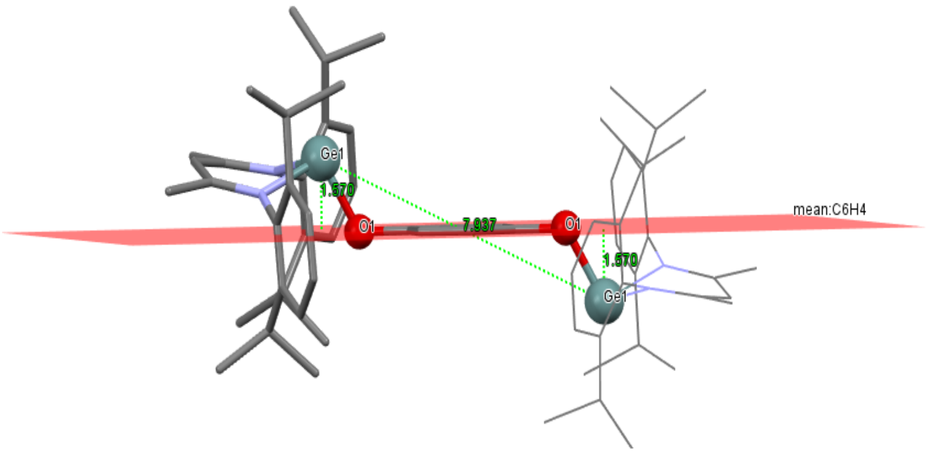

(**2**)

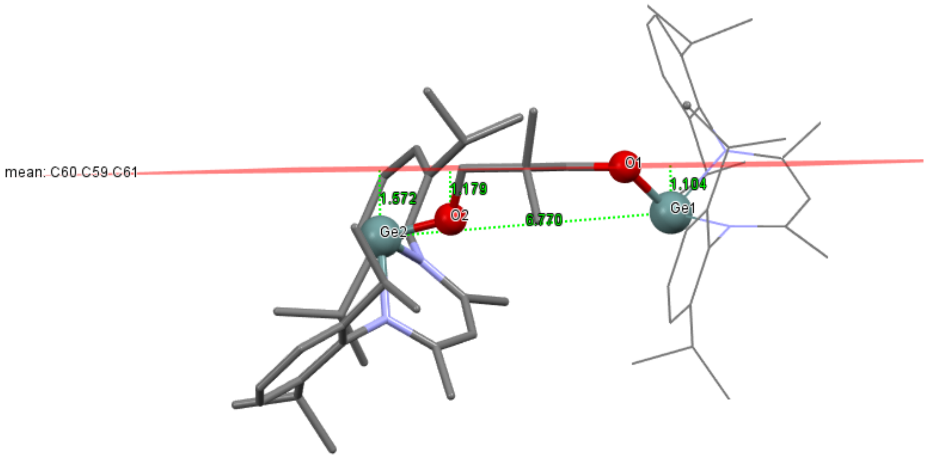

(**3**)

Fig. S1a Deviation of Ge atoms from the bridging plane of **1-3**

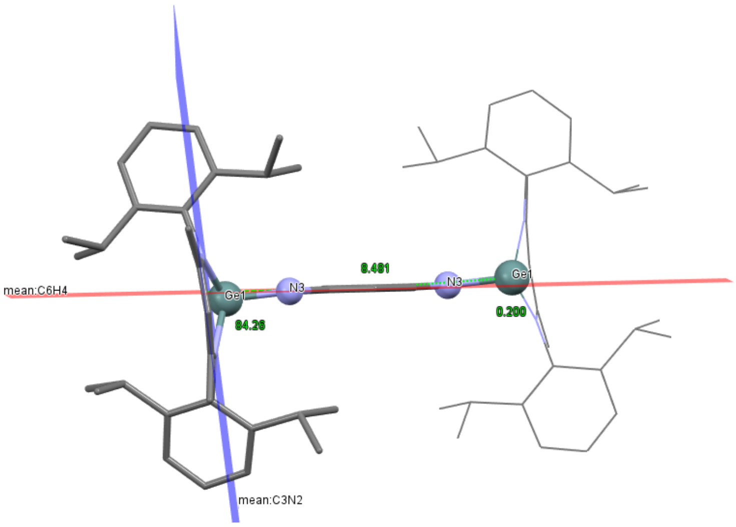

(4)

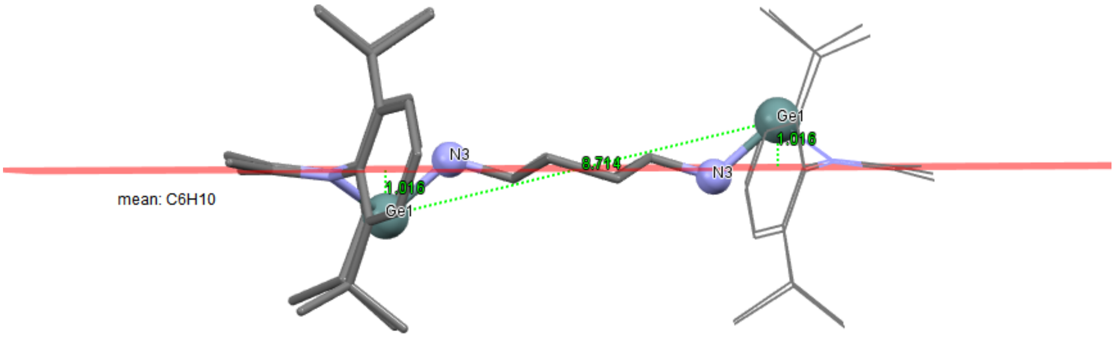

(5)

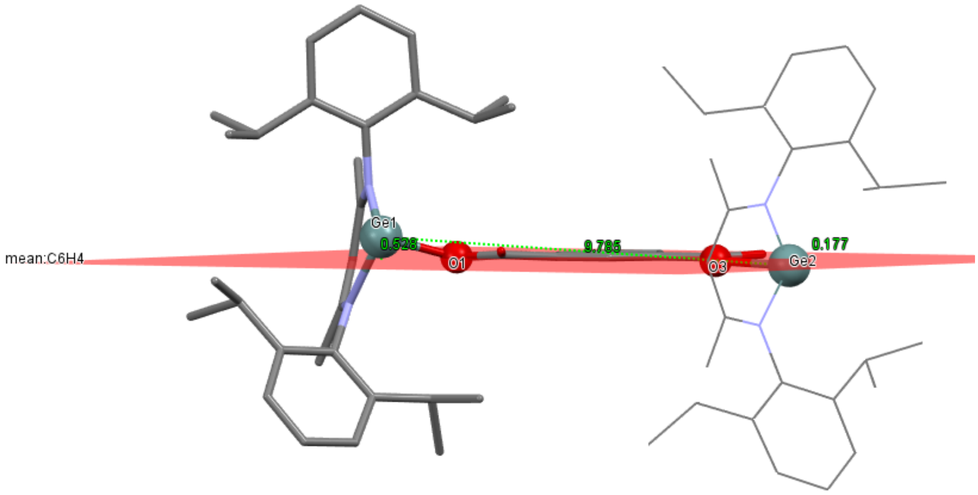

(6)

Fig. S1b Deviation of Ge atoms from the bridging plane of **4-6**

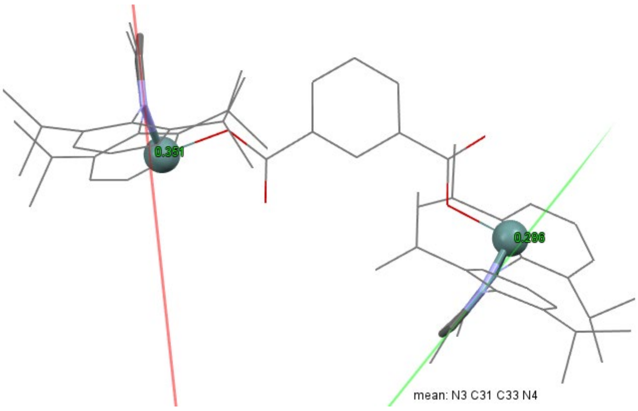

Fig. S2 Deviation of Ge atoms from the respective C_2N_2 planes of **6**

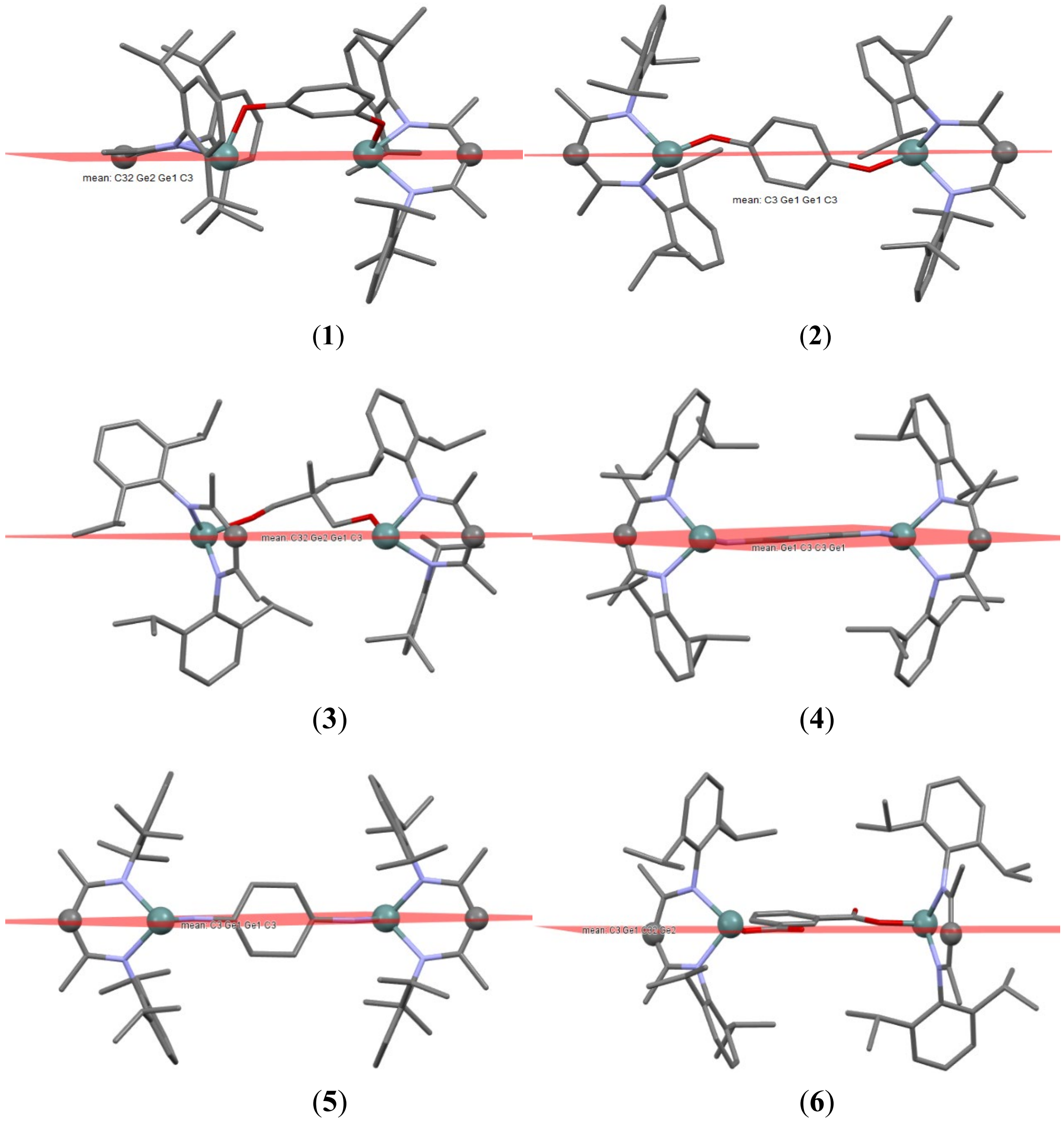

Fig. S3 Calculated planes over γ-C and Ge atoms of **1-6**

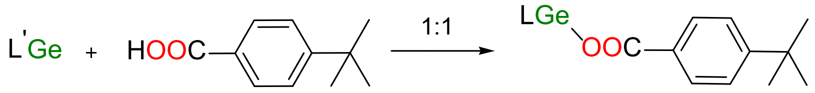

7

Scheme S1 Synthesis of **7**

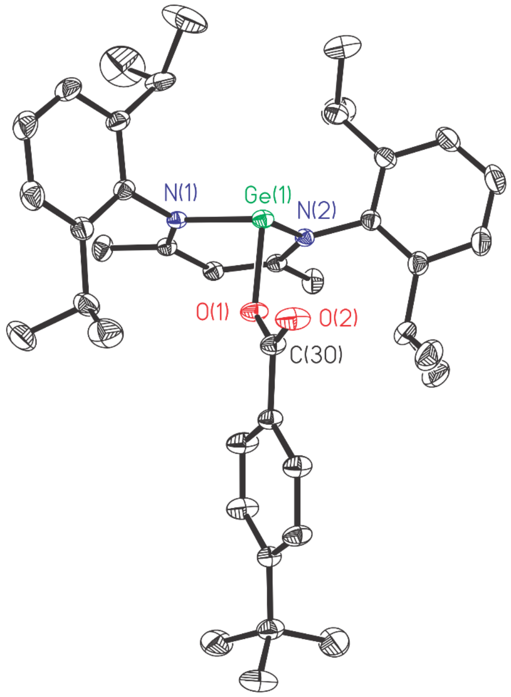

Fig. S4 Molecular structure of **7**

Molecular structure of **7** with the anisotropic displacement parameters depicted at the 30% probability level. The H atom are omitted for clarity. Selected bond lengths (Å) and angles (deg): Ge(1)–Cu(1) 2.2559(7), Ge(2)–Cu(2) 2.3383(7); Ge(1)–Cu(1)–Cu(2) 133.35 (3), Ge(1)–Cu(1)–O(3) 172.8(1).

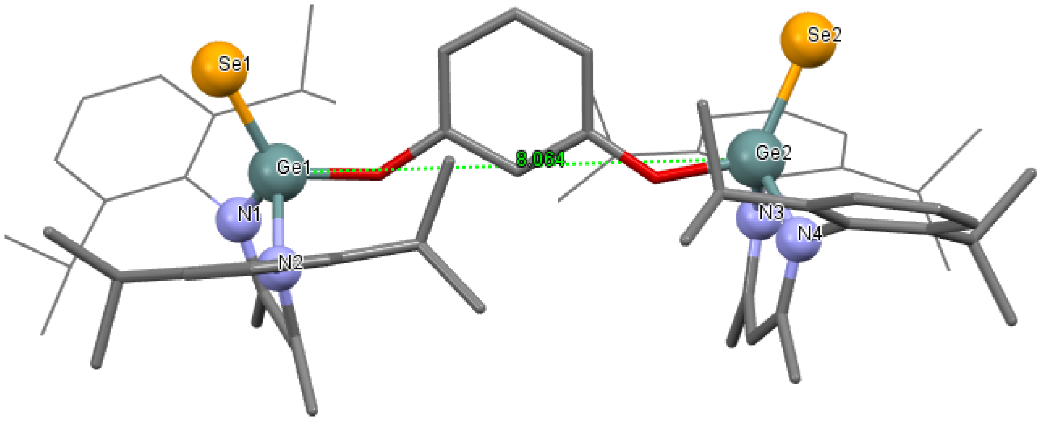

Fig. S5 Ge···Ge distance in **9**

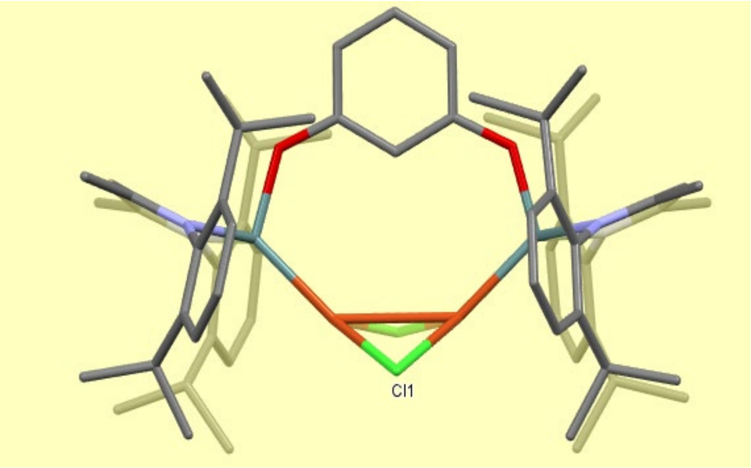

(**10**)

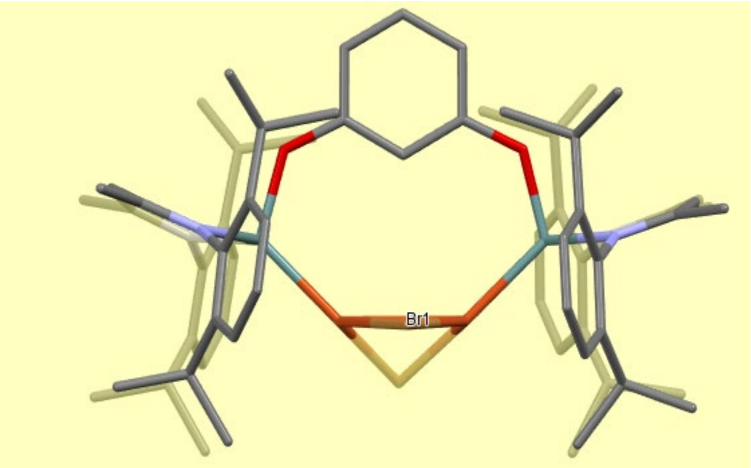

(**11**)

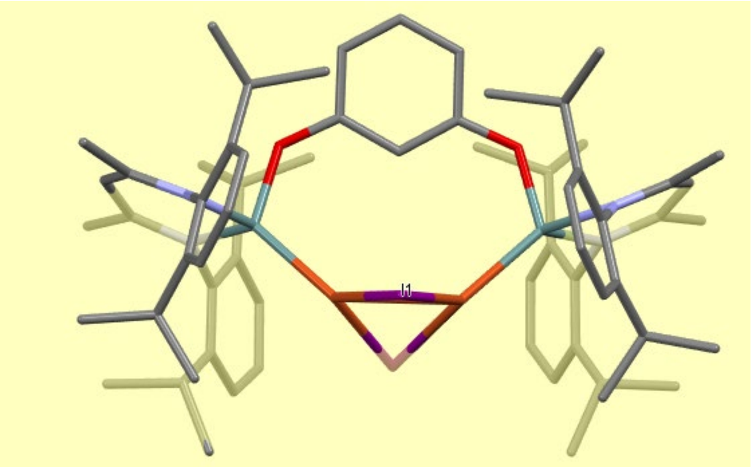

(**12**)

Fig. S6 Isostructural feature of **10-12**

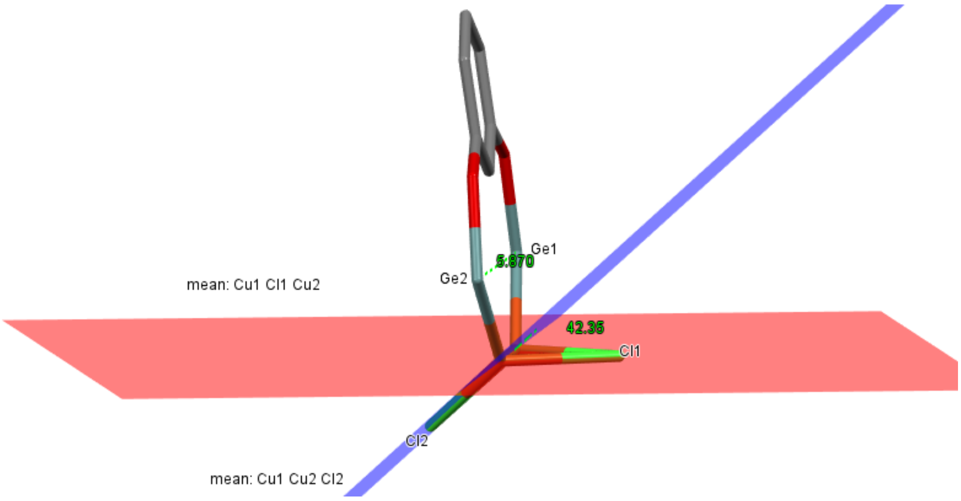

(**10**)

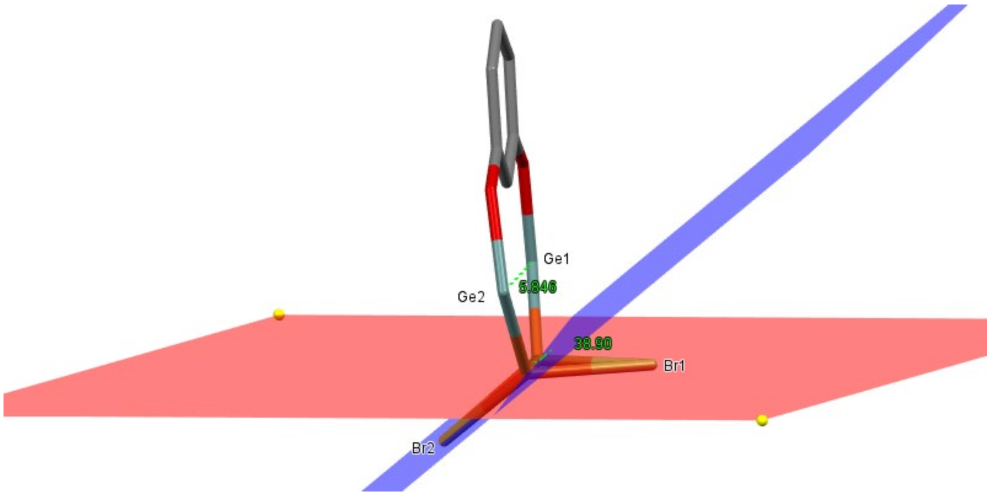

(**11**)

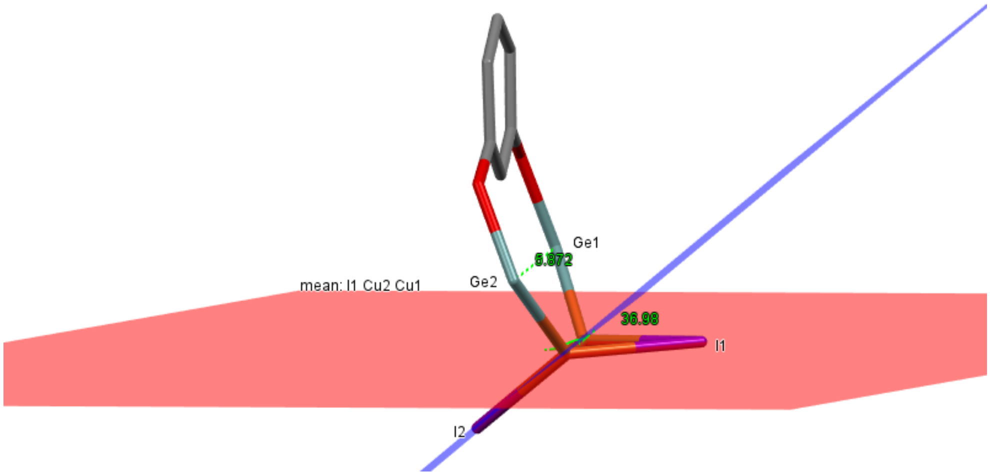

(**12**)

Fig. S7 Dihedral angles between Cu_2X planes of **10-12**

Table S1 Selected distances, bond lengths (Å) and bond angles (deg) of **10-12**

**Items**	**10**	**11**	**12**
Ge···Ge	5.870	5.846	5.872
Cu–Cu	2.6196(6)	2.5852(8)	2.5916(17)
Ge–Cu	2.2567(6)<br>2.2550(6)	2.2643(7)<br>2.2677(7)	2.2827(16)<br>2.2969(17)
Cu–X	2.2432(12)<br>2.2629(12)<br>2.3129(12)<br>2.3603(13)	2.3799(9)<br>2.3809(8)<br>2.4451(8)<br>2.4474(9)	2.5555(17)<br>2.5677(15)<br>2.5766(18)<br>2.5768(16)
Cu–X–Cu	68.18(4)<br>71.09(4)	63.79(2)<br>65.78(2)	60.50(4)<br>60.66(4)
Cu_2X dihedral angle	42.35	38.90	36.98

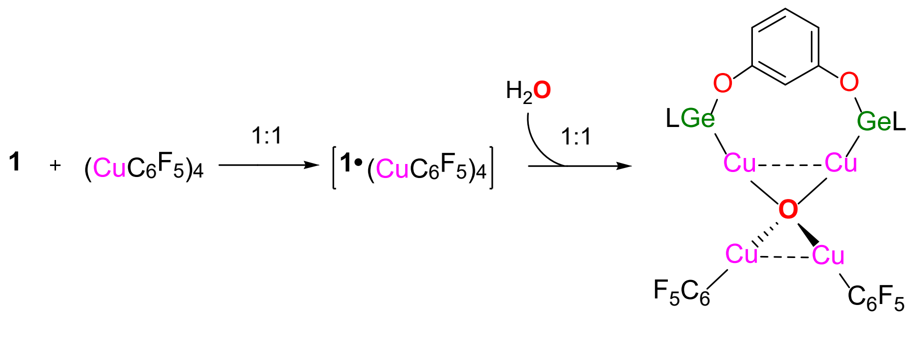

13

Scheme S2 Reaction of **1** with $(CuC_6F_5)_4$

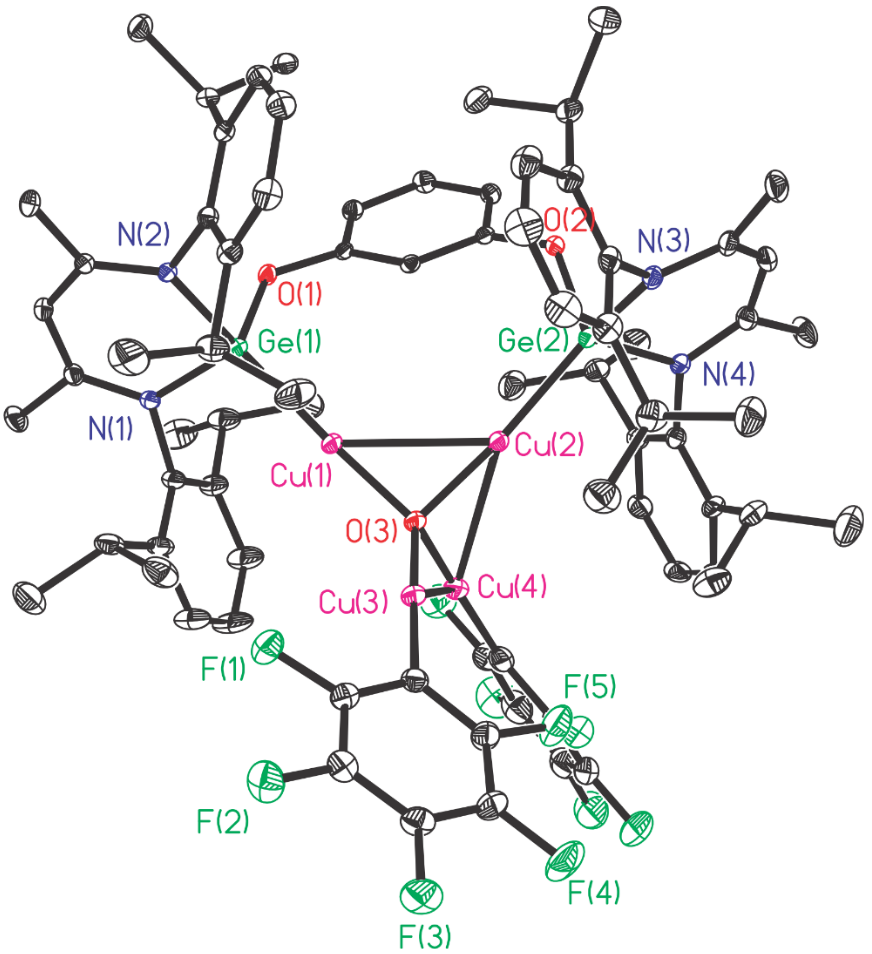

Fig. S8 Molecular structure of **13**

Molecular structure of **13** with the anisotropic displacement parameters depicted at the 30% probability level. The H atom are omitted for clarity Selected bond lengths (Å) and angles (deg): Ge(1)–Cu(1) 2.2554(5), Ge(2)–Cu(2) 2.2378(5), Cu(1)–Cu(2) 2.6867(6), Cu(2)–Cu(4) 2.8003(7), Cu(3)–Cu(4) 2.8328(8); Ge(1)–Cu(1)–Cu(2) 133.35 (2), Ge(1)–Cu(1)–O(3) 172.99(9).

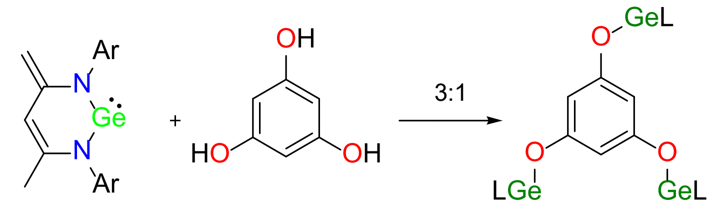

L'Ge 14

Scheme S3 Synthesis of trigermylene

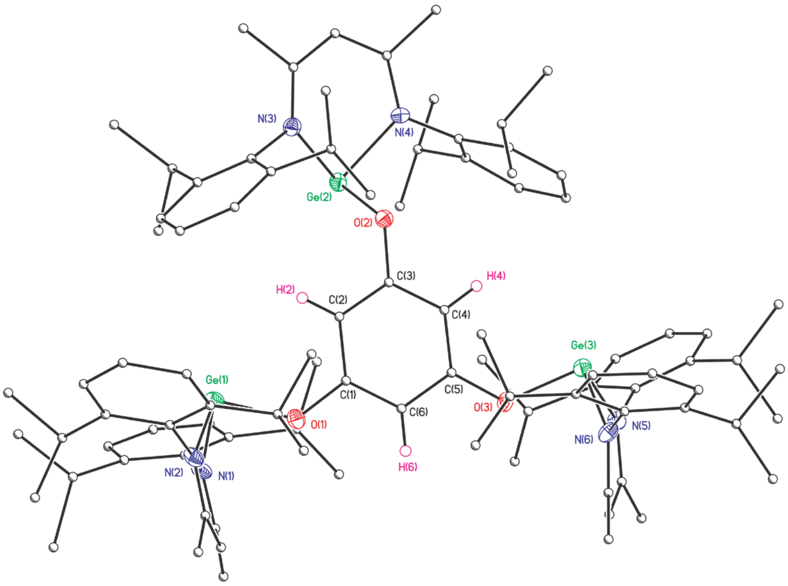

Fig. S9 Molecular structure of trigermylene **14**

Molecular structure of **15** with the anisotropic displacement parameters depicted at the 30% probability level. The H atom are omitted for clarity Selected bond lengths (Å) and angles (deg): Ge(1)–O(1) 1.850(8), Ge(2)–O(2) 1.827(7), Ge(3)–O(3) 1.865(7); Ge(1)–O(1)–C(1) 124.5(7), Ge(2)–O(2)–C(3) 119.0(6), Ge(3)–O(3)–C(5) 123.0(6).

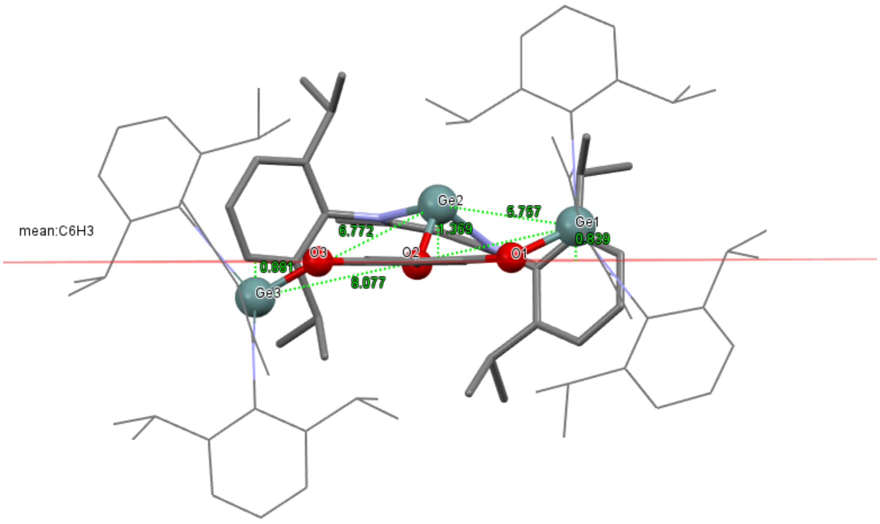

Fig. S10 Deviation of Ge atoms from the bridging plane of **14**

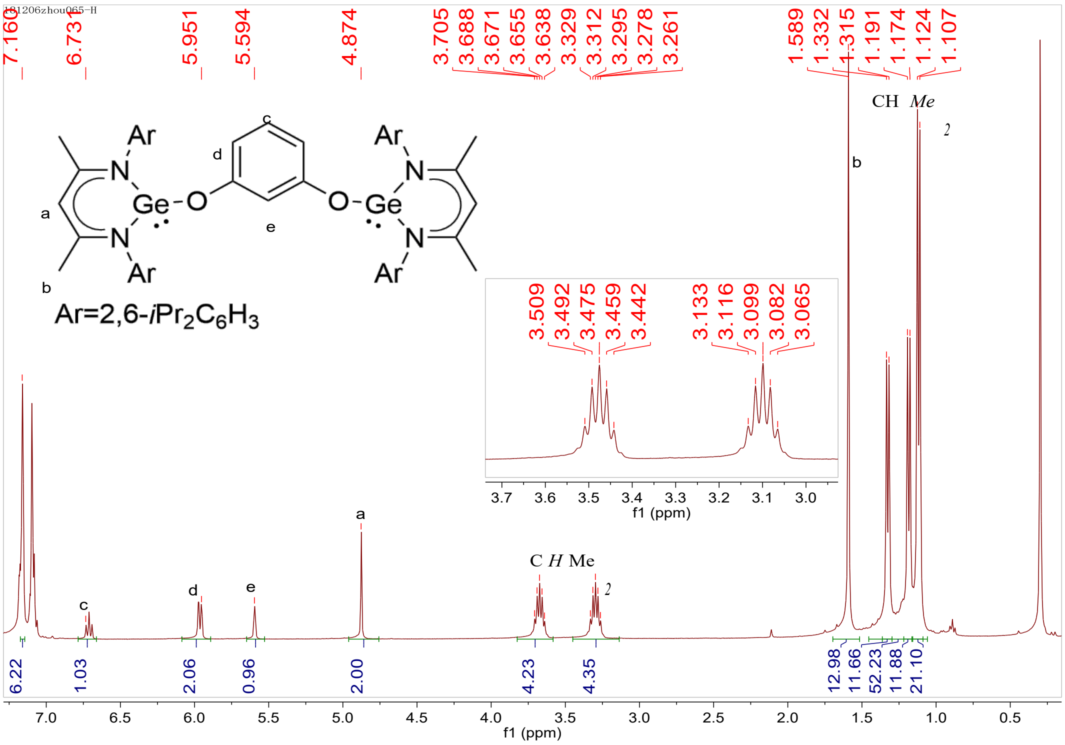

Fig. S11 The ^{1}H NMR spectrum of 1,3-$C_6H_4(OGeL)_2$ (**1**)

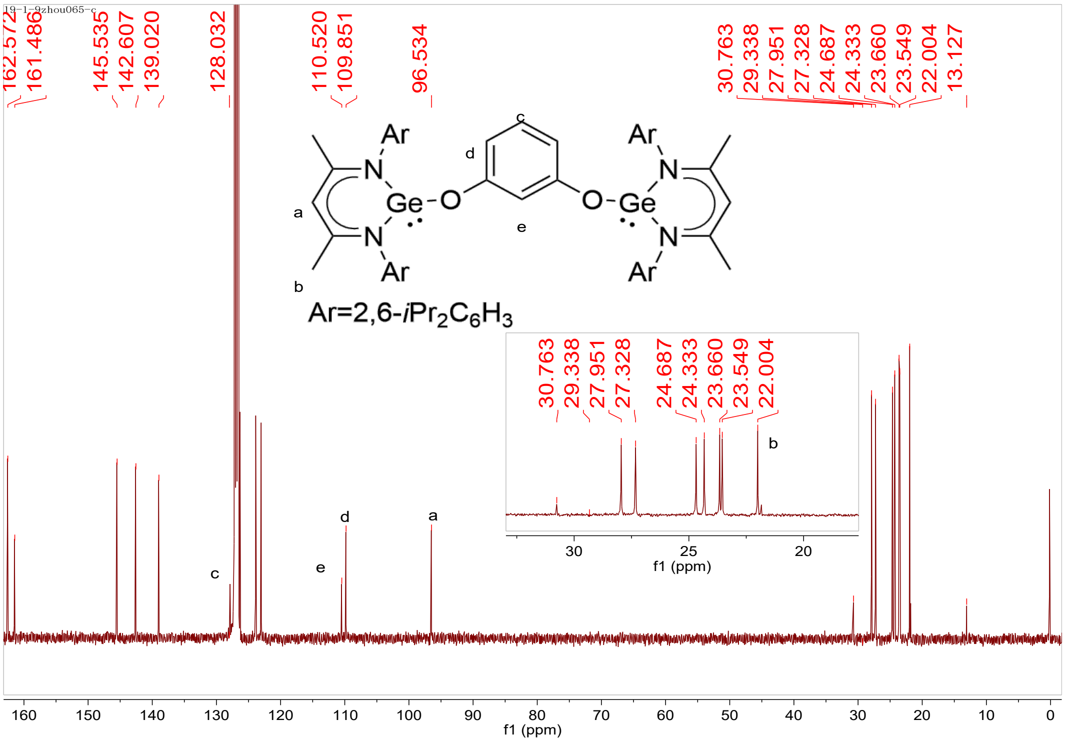

Fig. S12 The ^{13}C NMR spectrum of 1,3-$C_6H_4(OGeL)_2$ (**1**)

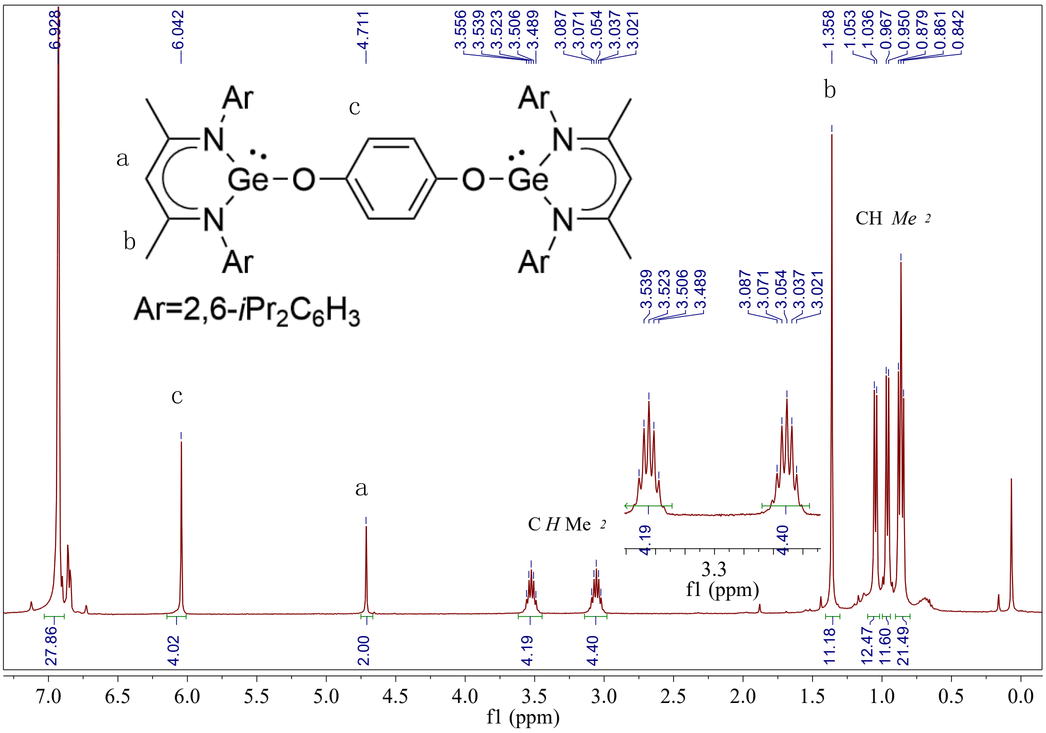

Fig. S13 The ^{1}H NMR spectrum of 1,4-$C_6H_4(OGeL)_2$ (**2**)

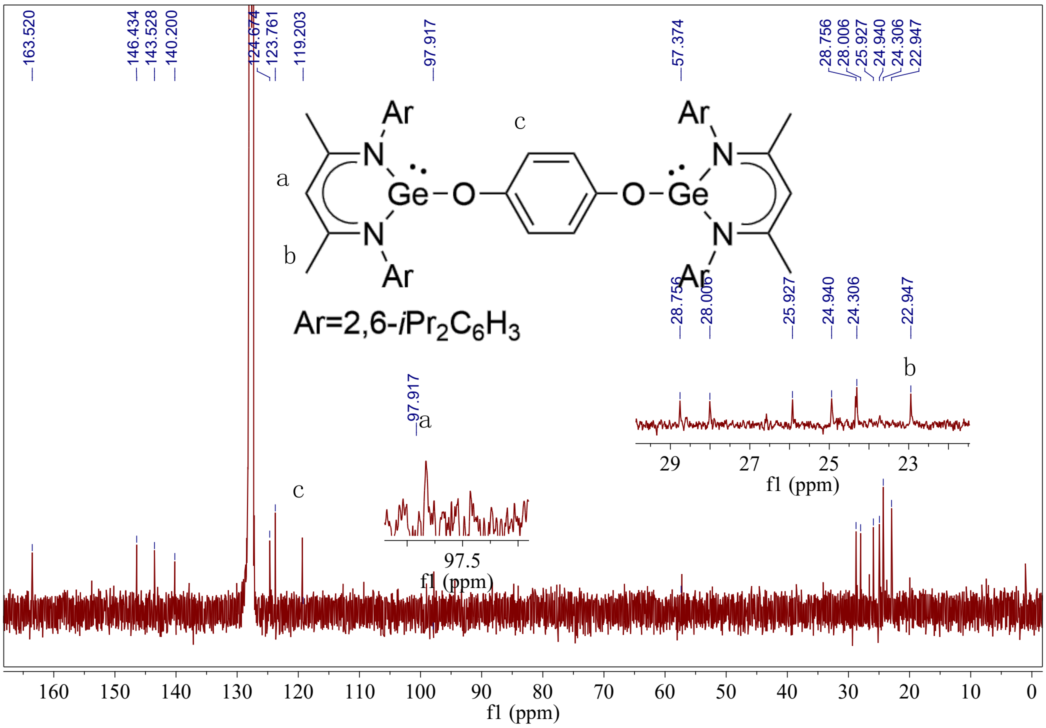

Fig. S14 The ^{13}C NMR spectrum of 1,4-$C_6H_4(OGeL)_2$ (**2**)

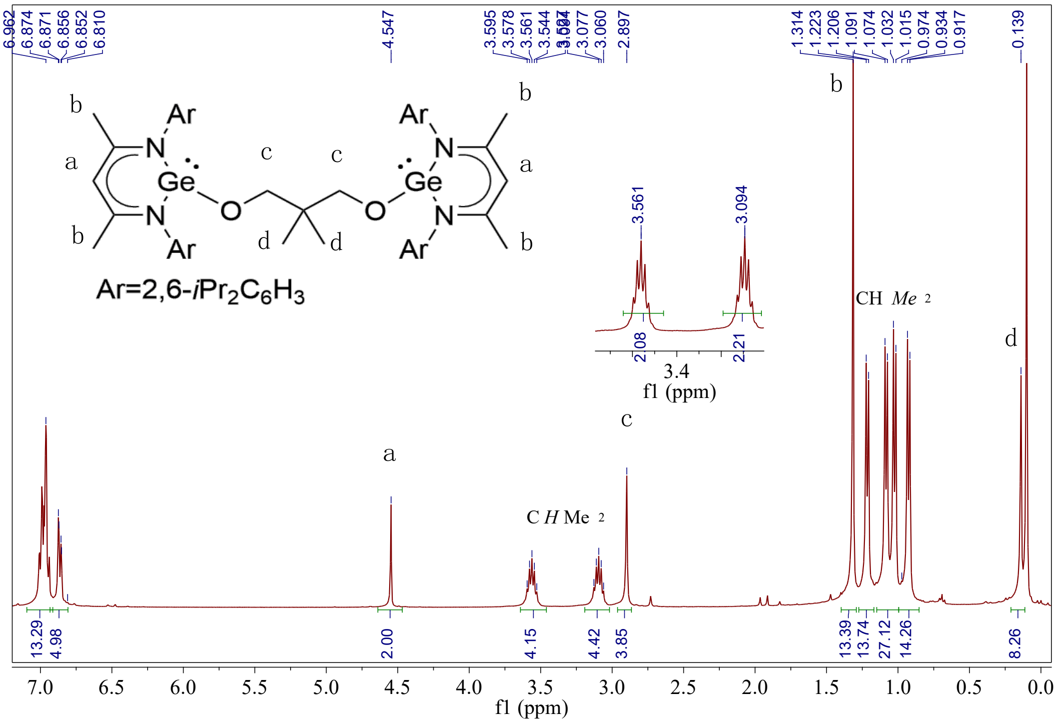

Fig. S15 The ^{1}H NMR spectrum of $Me_2C(CH_2OGeL)_2$ (**3**)

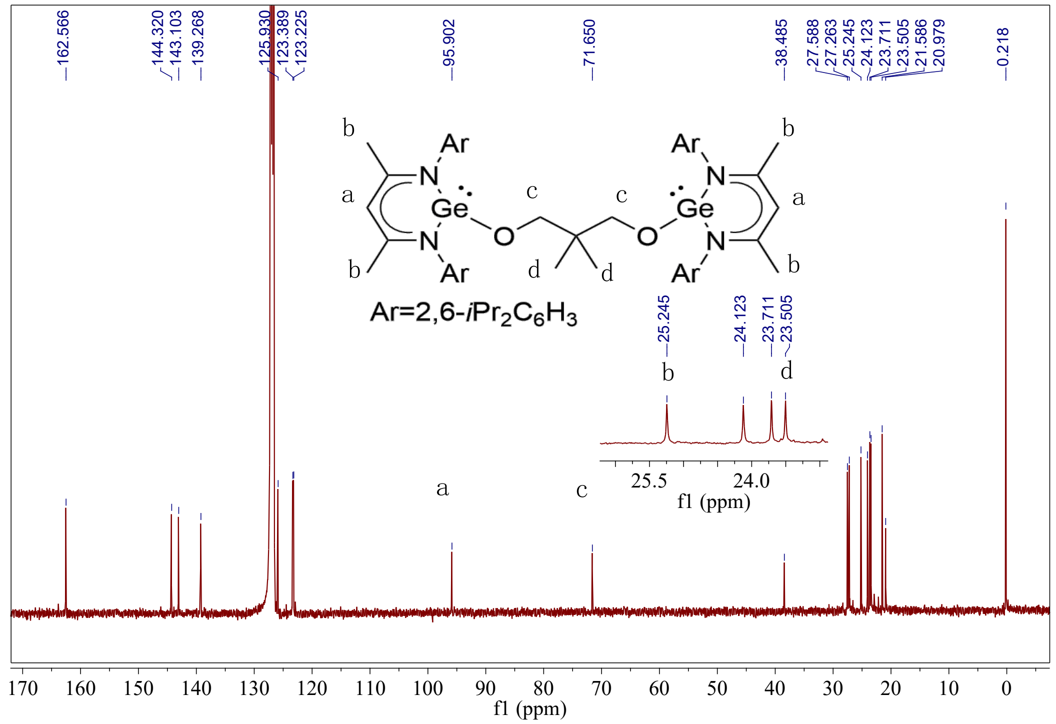

Fig. S16 The ^{13}C NMR spectrum of $Me_2C(CH_2OGeL)_2$ (**3**)

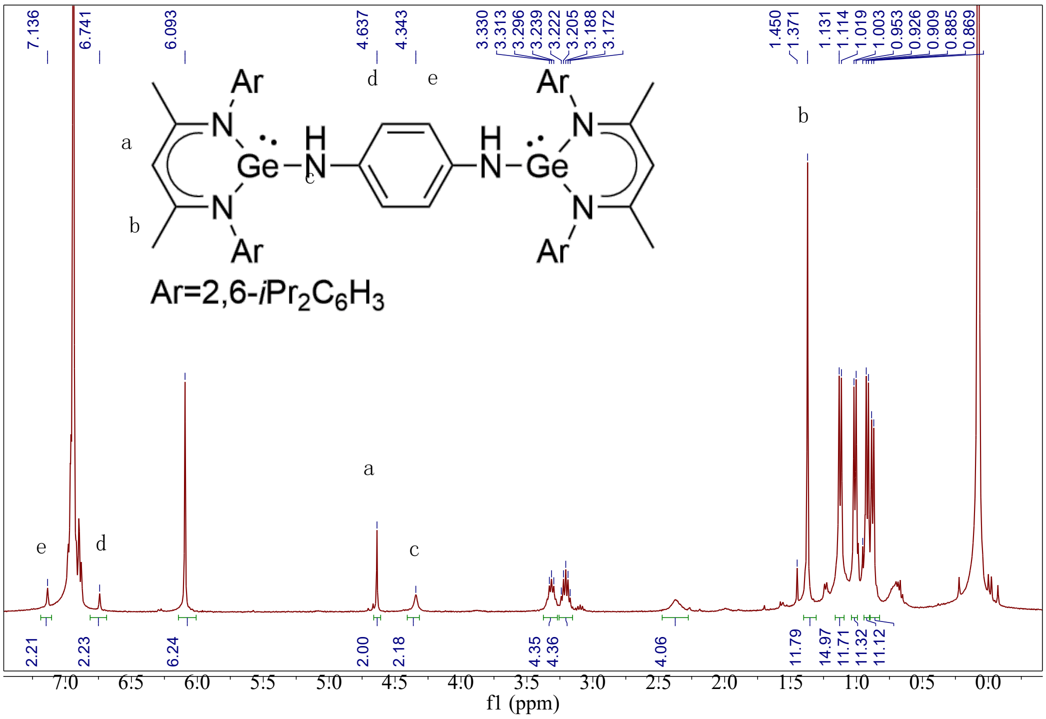

Fig. S17 The ^{1}H NMR spectrum of 1,4-$C_6H_4[N(H)GeL]_2$ (**4**)

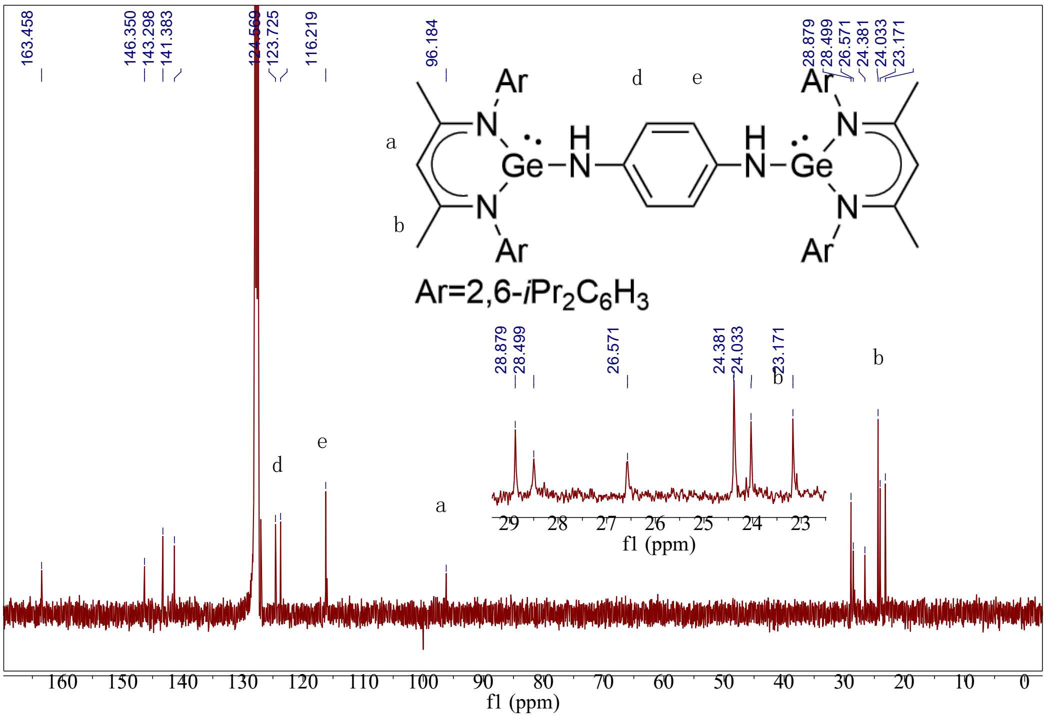

Fig. S18 The ^{13}C NMR spectrum of 1,4-$C_6H_4[N(H)GeL]_2$ (**4**)

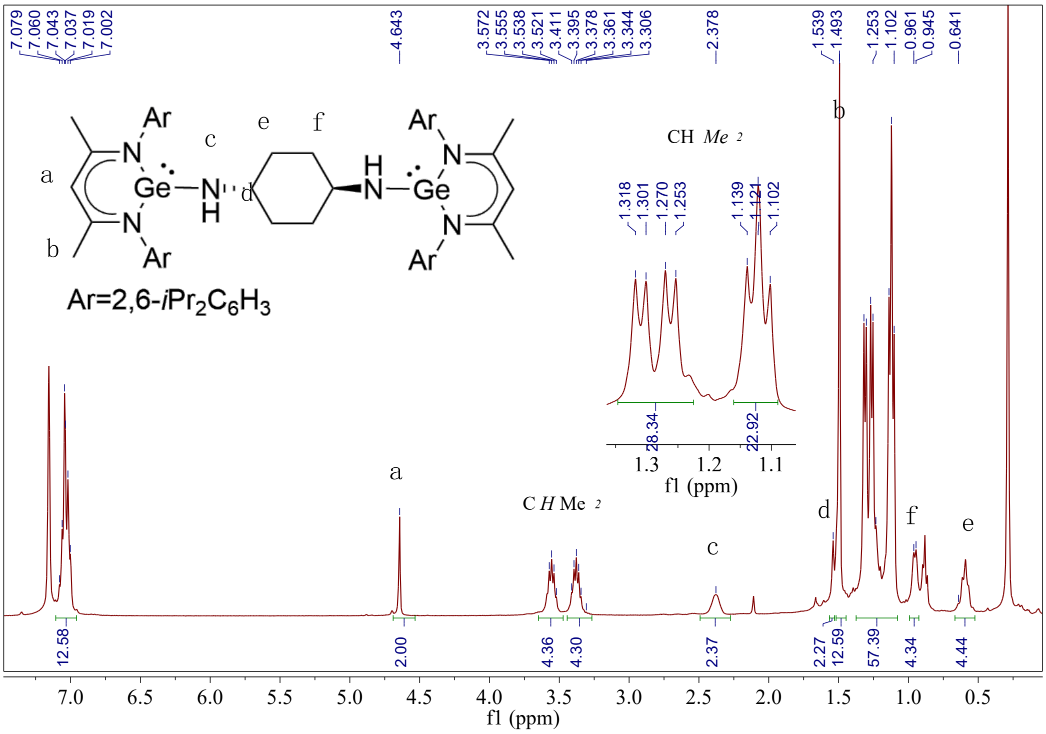

Fig. S19 The ^{1}H NMR spectrum of 1,4-C_6H_{10}[N(H)GeL]$_2$ (**5**)

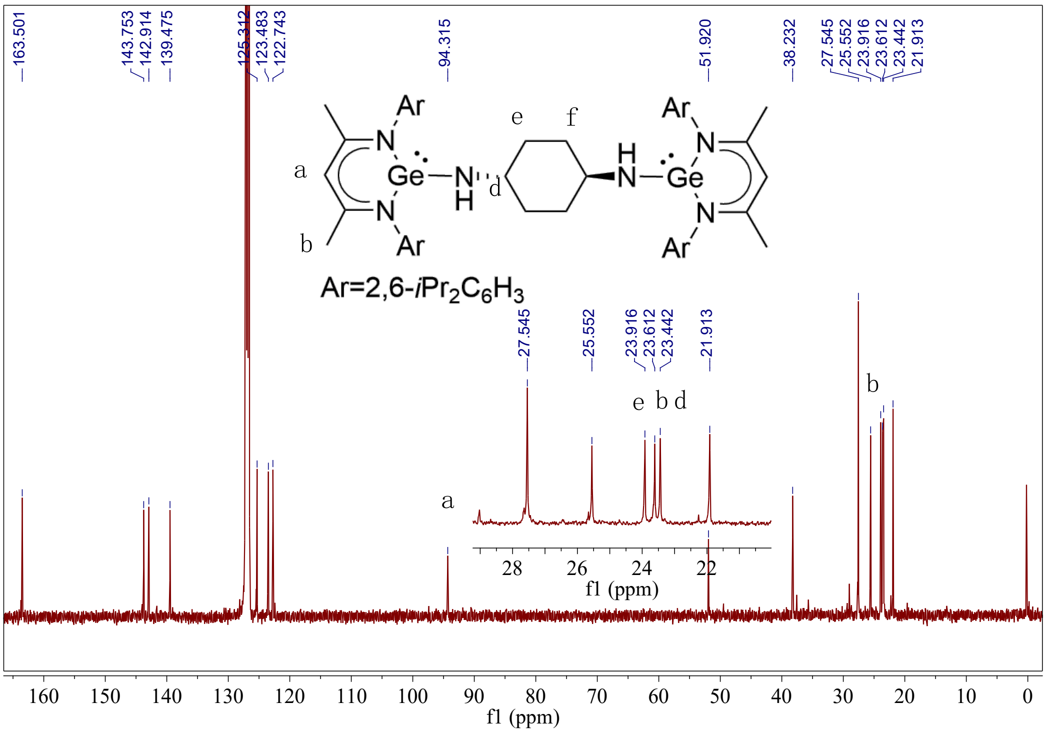

Fig. S20 The ^{13}C NMR spectrum of 1,4-C_6H_{10}[N(H)GeL]$_2$ (**5**)

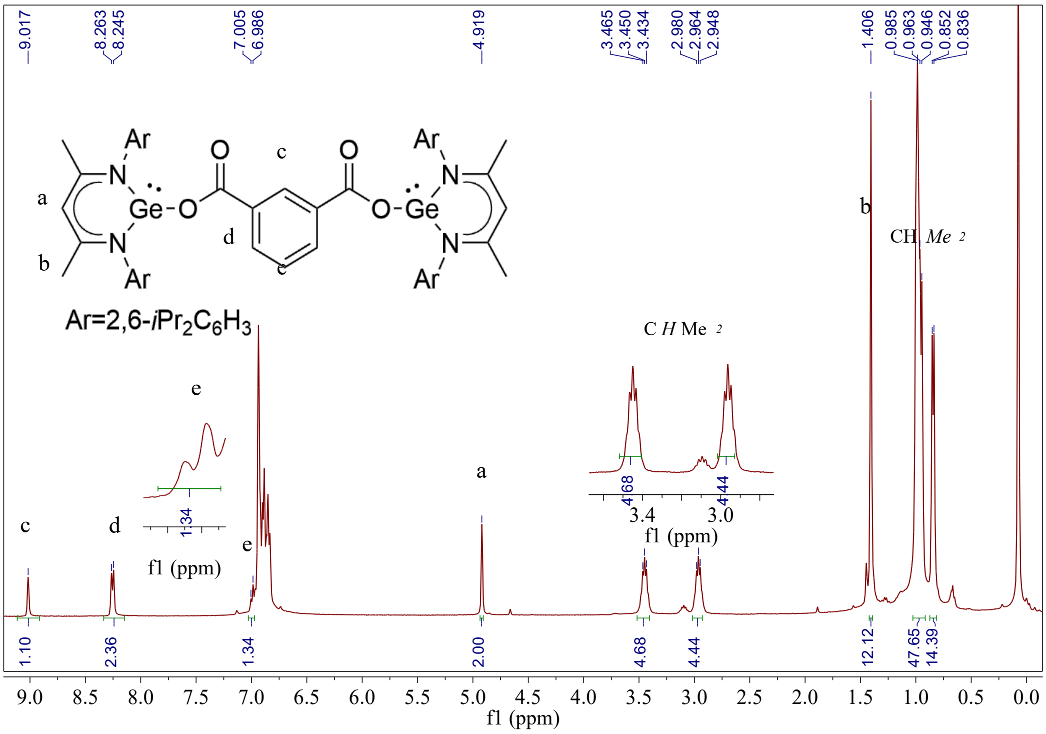

Fig. S21 The ^{1}H NMR spectrum of 1,3-$C_6H_4[C(O)OGeL]_2$ (**6**)

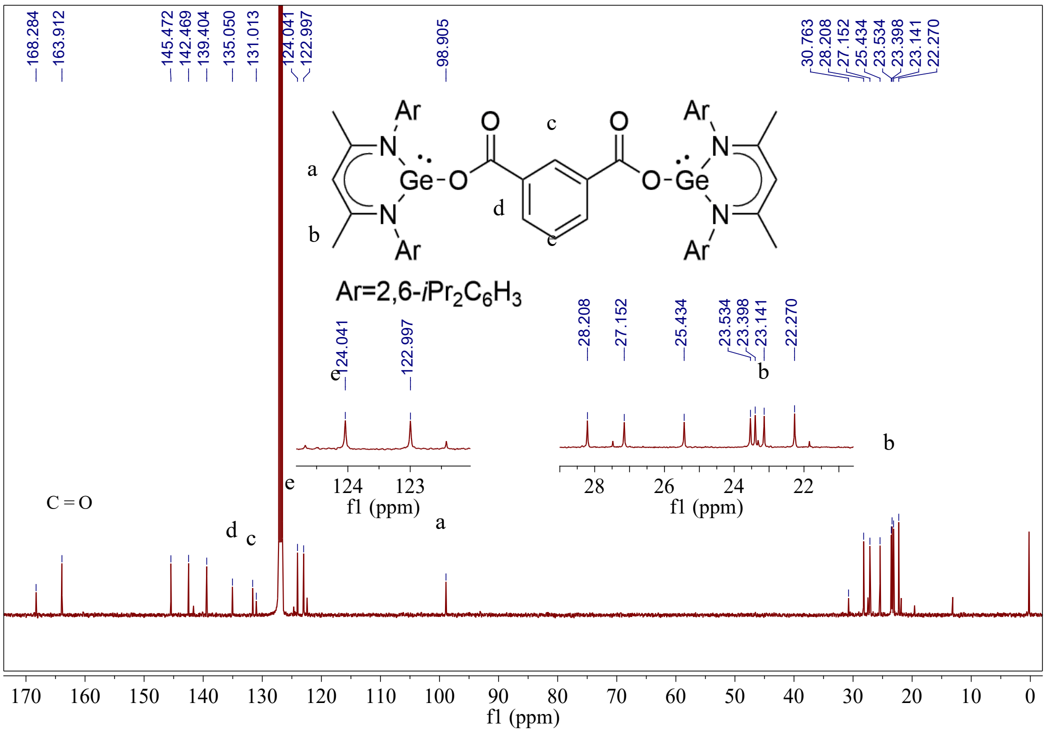

Fig. S22 The ^{13}C NMR spectrum of 1,3-$C_6H_4[C(O)OGeL]_2$ (**6**)

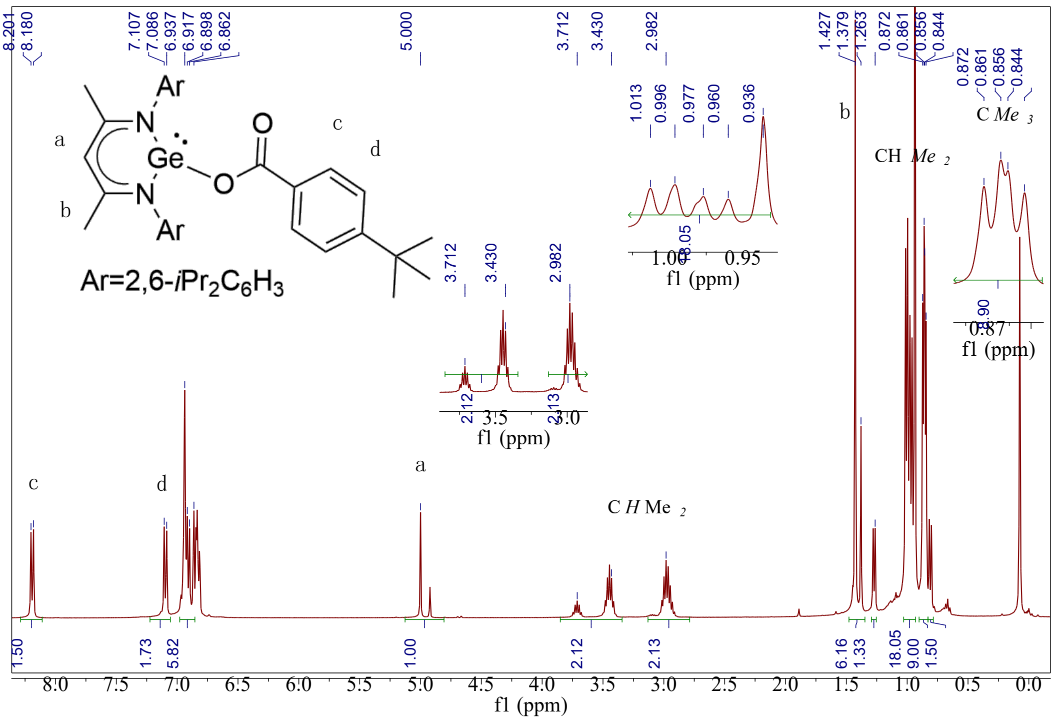

Fig. S23 The ^{1}H NMR spectrum of 4-*t*Bu-$C_6H_4C(O)OGeL$ (**7**)

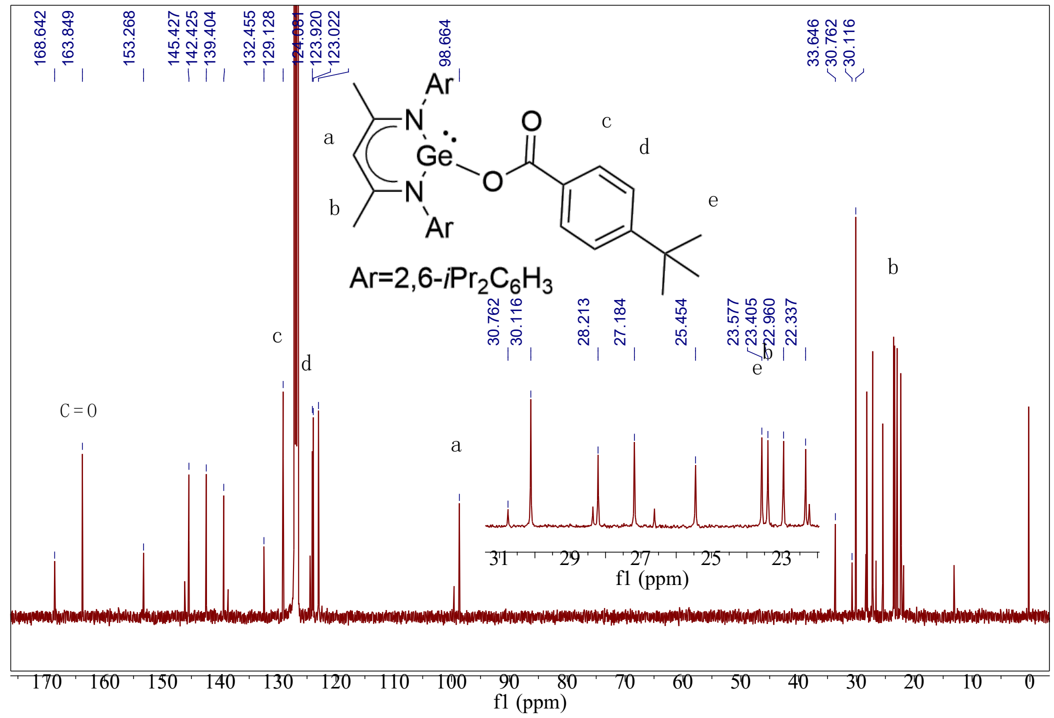

Fig. S24 The ^{13}C NMR spectrum of 4-*t*Bu-$C_6H_4C(O)OGeL$ (**7**)

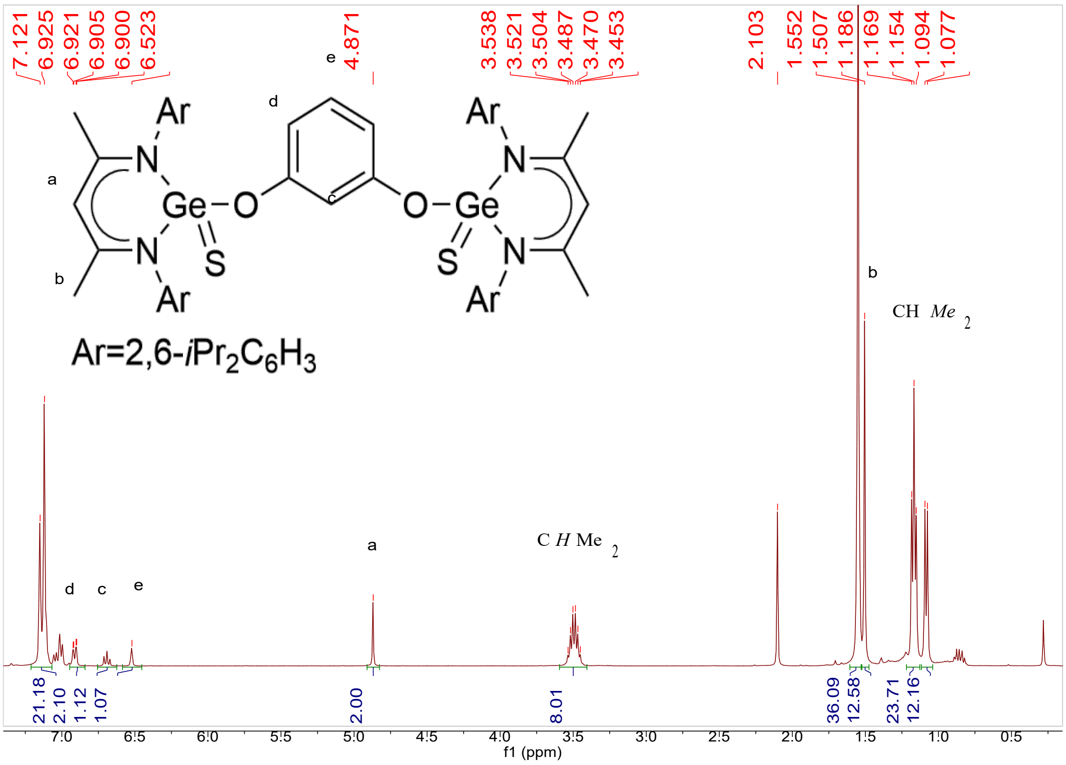

Fig. S25 The ^{1}H NMR spectrum of 1,3-$C_6H_4[OGe(S)L]_2$ (**8**)

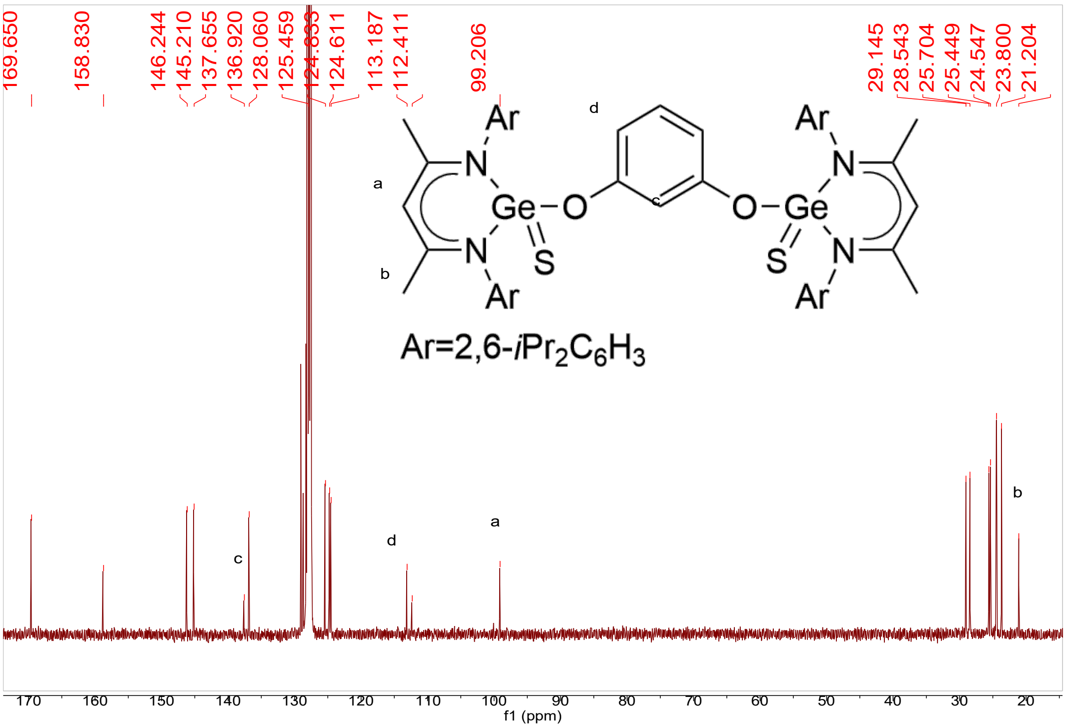

Fig. S26 The ^{13}C NMR spectrum of 1,3-$C_6H_4[OGe(S)L]_2$ (**8**)

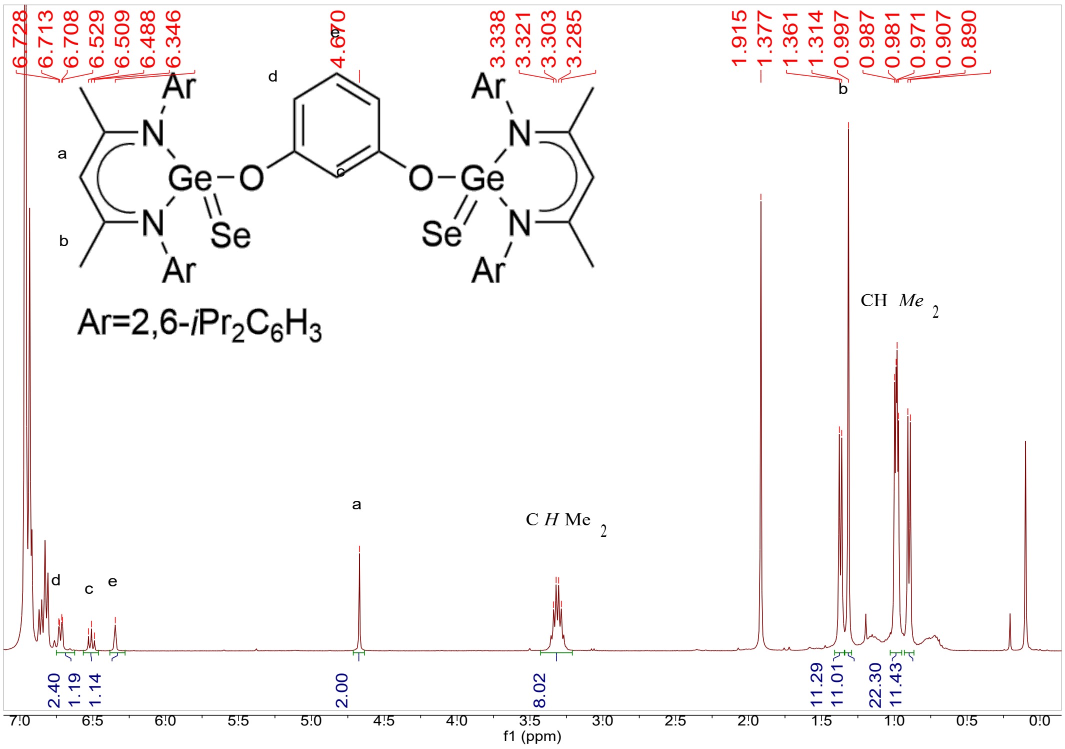

Fig. S27 The ^{1}H NMR spectrum of 1,3-$C_6H_4[OGe(Se)L]_2$ (**9**)

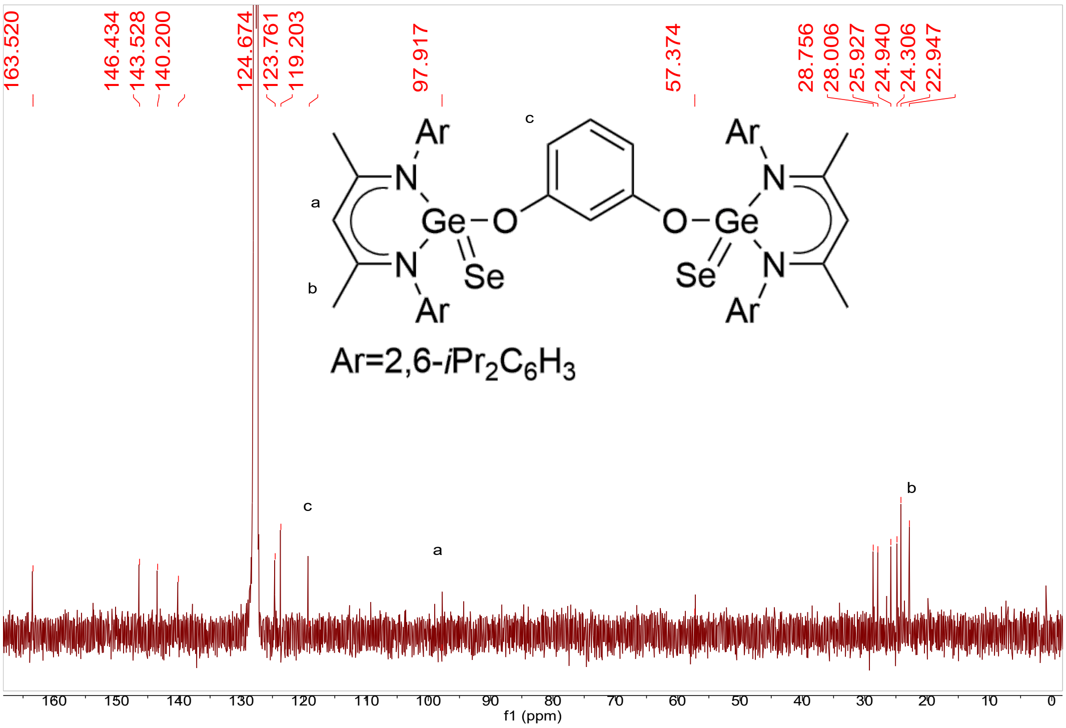

Fig. S28 The ^{13}C NMR spectrum of 1,3-$C_6H_4[OGe(Se)L]_2$ (**9**)

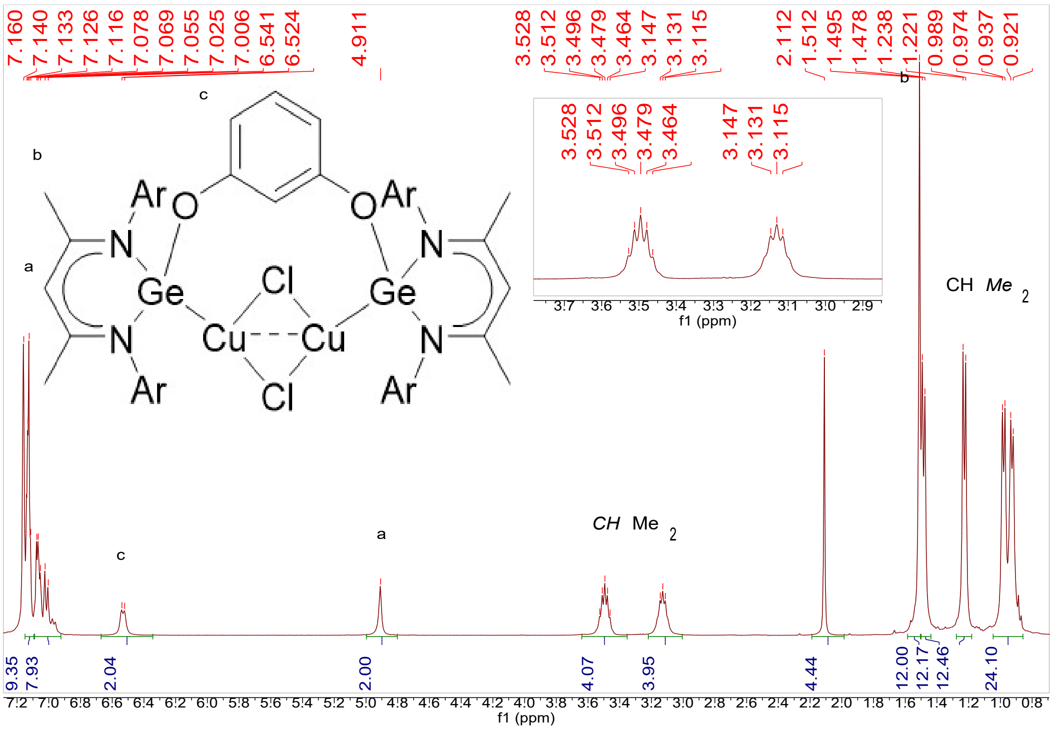

Fig. S29 The ^{1}H NMR spectrum of $(CuCl)_2[1,3\text{-}C_6H_4(OGeL)_2]$ (**10**)

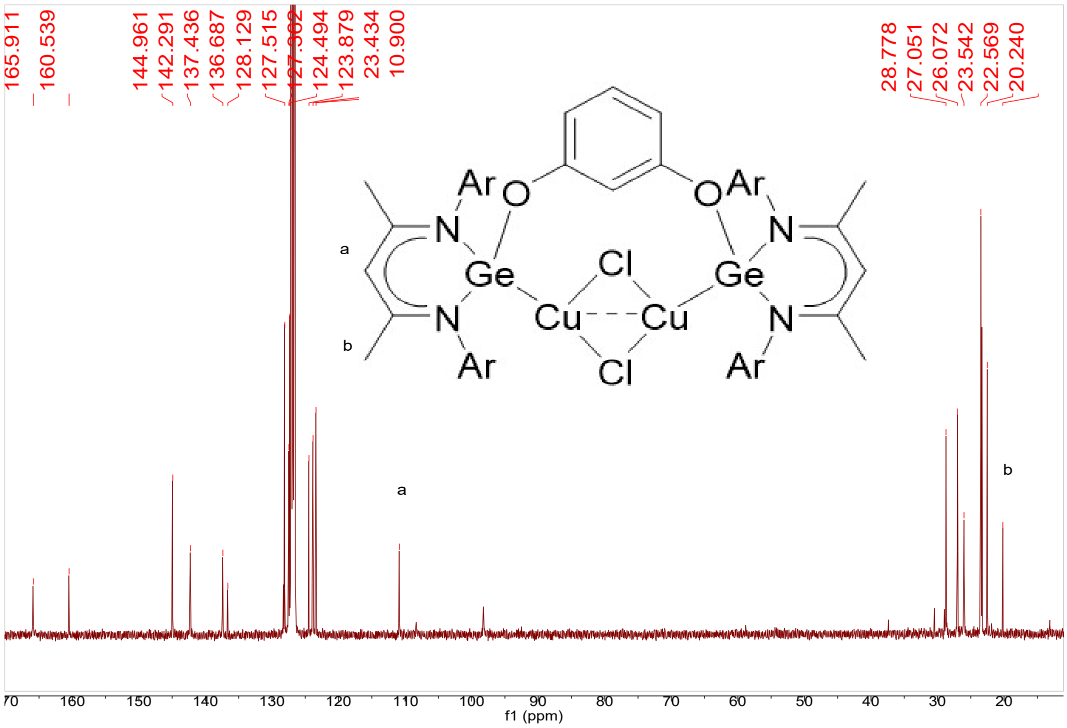

Fig. S30 The ^{13}C NMR spectrum of $(CuCl)_2[1,3\text{-}C_6H_4(OGeL)_2]$ (**10**)

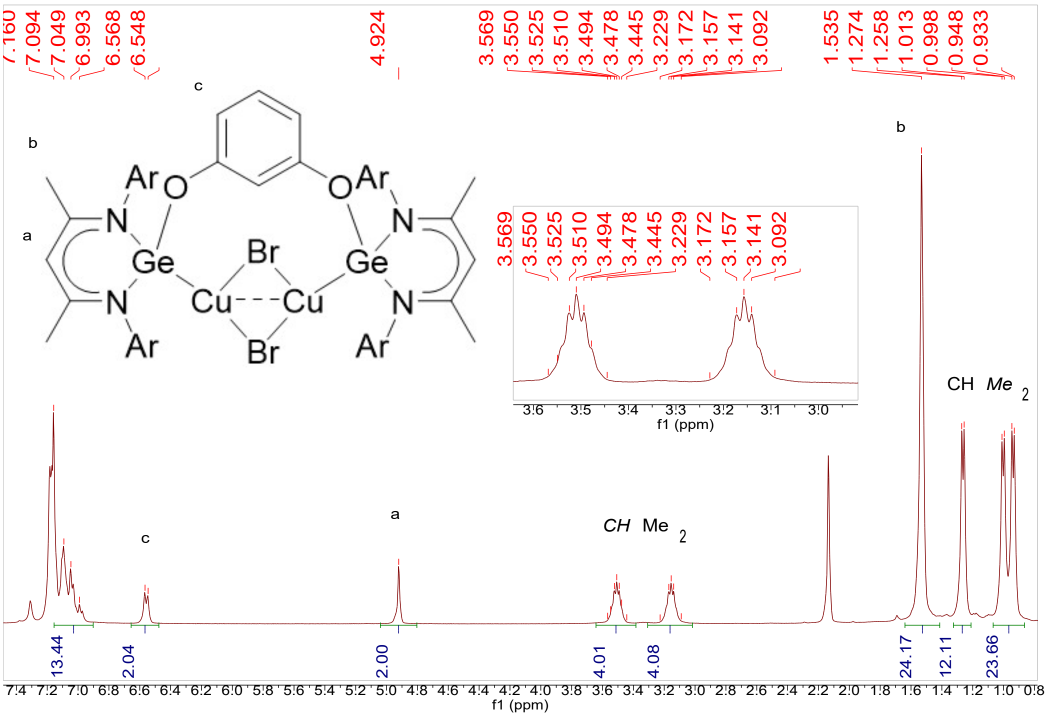

Fig. S31 The ^{1}H NMR spectrum of $(CuBr)_2[1,3\text{-}C_6H_4(OGeL)_2]$ (**11**)

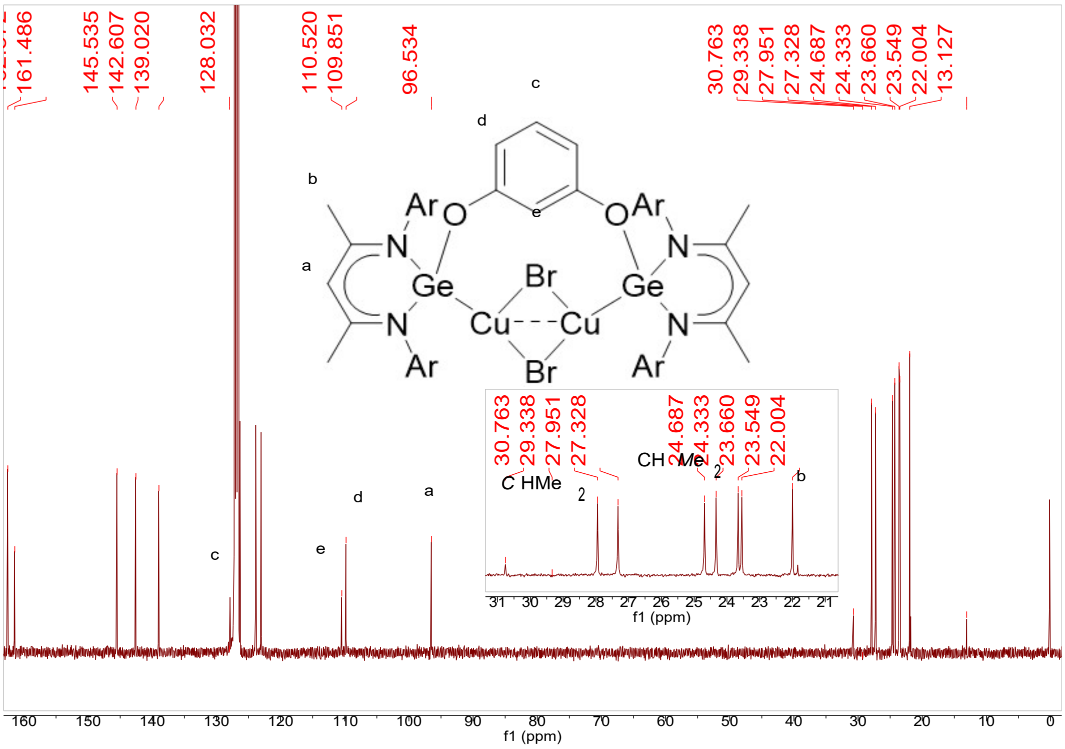

Fig. S32 The ^{13}C NMR spectrum of $(CuBr)_2[1,3\text{-}C_6H_4(OGeL)_2]$ (**11**)

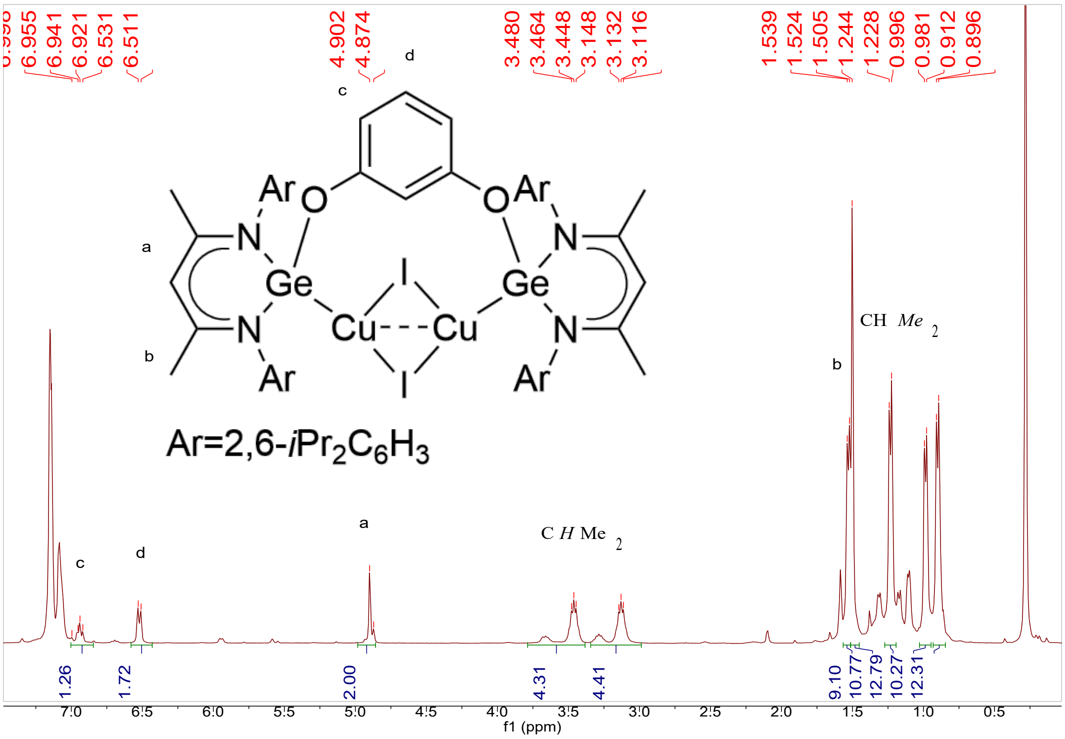

Fig. S33 The ^{1}H NMR spectrum of $(CuI)_2[1,3\text{-}C_6H_4(OGeL)_2]$ (**12**)

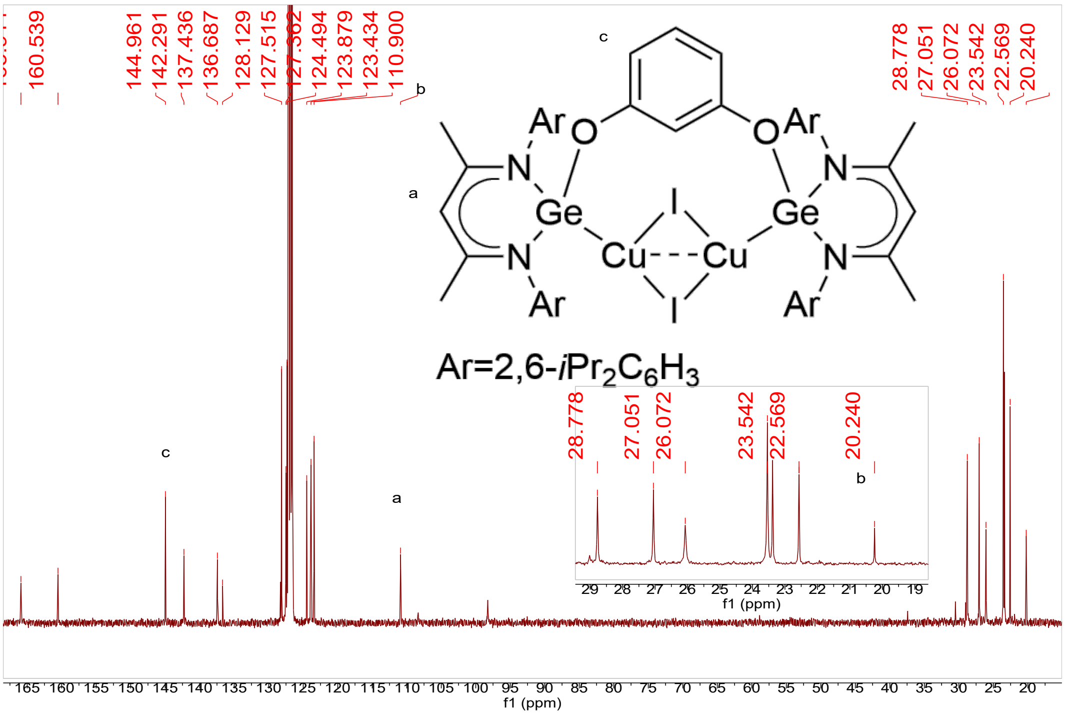

Fig. S34 The ^{13}C NMR spectrum of $(CuI)_2[1,3\text{-}C_6H_4(OGeL)_2]$ (**12**)

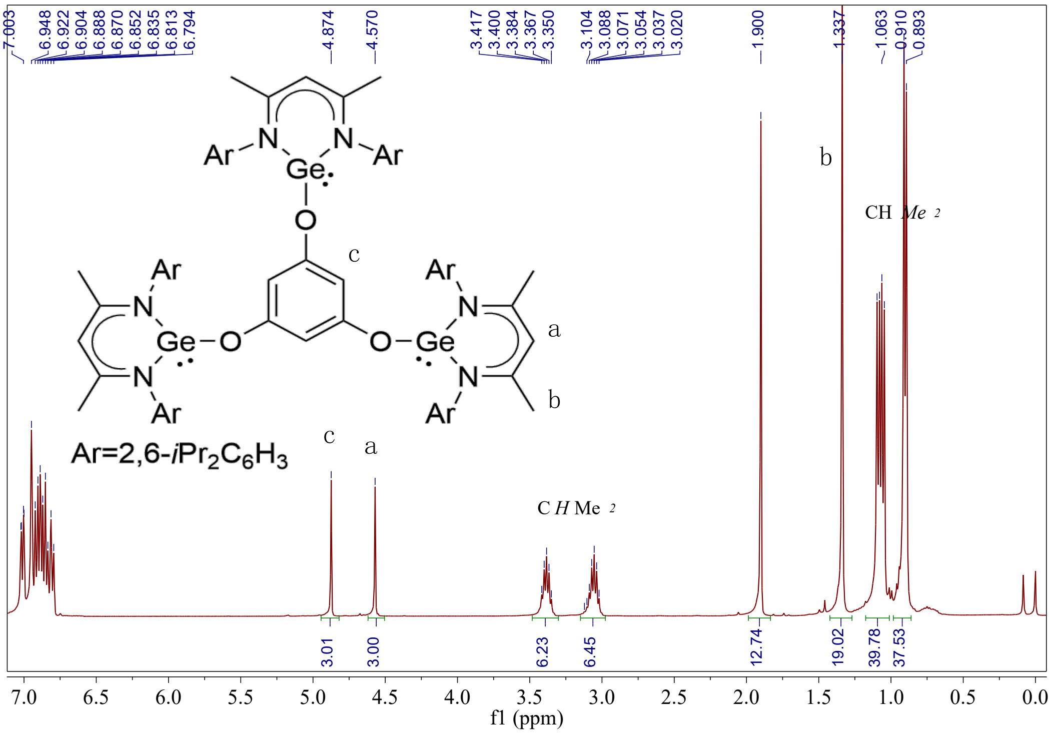

Fig. S35 The ^{1}H NMR spectrum of 1,3,5-$C_6H_3(OGeL)_3$ (**14**)

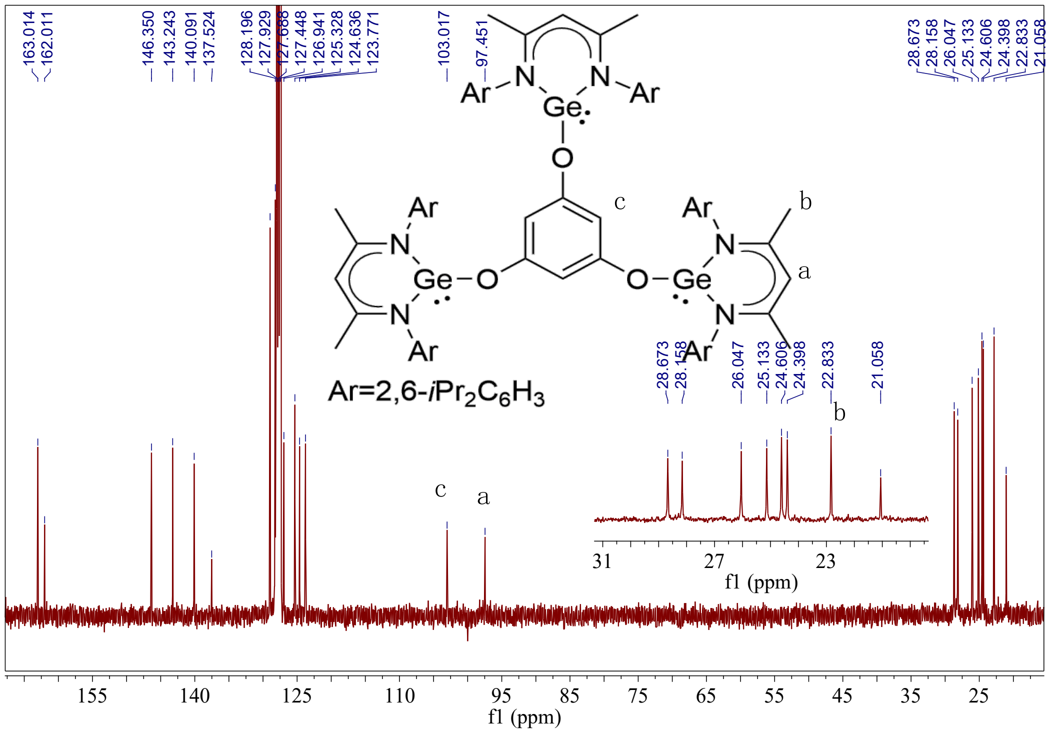

Fig. S36 The ^{13}C NMR spectrum of 1,3,5-$C_6H_3(OGeL)_3$ (**14**)

Table S2a Crystal data and structure refinements for **1-7**

Identification code	**1**	**2**	**3**	**4**	**5**	**6**	**7**
CCDC number	2151189	2151190	2151191	2151192	2151193	2151194	2151195
Formula	$C_{64}H_{86}Ge_2N_4O_2$	$C_{32}H_{43}GeN_2O$	$C_{63}H_{92}Ge_2N_4O_2$	$C_{64}H_{88}Ge_2N_6$	$C_{64}H_{94}Ge_2N_6$	$C_{66}H_{86}Ge_2N_4O_4$	$C_{40}H_{54}GeN_2O_2$
Formula weight	1088.54	544.27	1082.58	1086.58	1092.63	1144.56	667.44
Temperature/K	296(2)	296(2)	296(2)	296(2)	296(2)	296(2)	296(2)
Crystal system	triclinic	monoclinic	triclinic	triclinic	monoclinic	monoclinic	triclinic
Space group	P-1	$P2_1/c$	P-1	P-1	$P2_1/c$	$P2_1/n$	P-1
a/Å	12.161(2)	11.8671(2)	12.230(3)	7.0514(6)	12.961(3)	21.7688(14)	12.2935(3)
b/Å	12.266(3)	14.7518(2)	16.749(4)	12.3898(11)	20.079(4)	12.7410(8)	12.9494(3)
c/Å	24.792(5)	17.2409(3)	17.857(4)	19.9038(17)	15.459(3)	23.0876(15)	14.9159(4)
α/°	77.33(3)	90	113.104(12)	91.955(5)	90	90	98.6040(10)
β/°	85.58(3)	100.5560(10)	97.624(11)	95.915(4)	104.347(13)	95.144(4)	111.8160(10)
γ/°	61.07(3)	90	108.716(12)	98.946(5)	90	90	111.7850(10)
Volume/Å^3	3156.2(14)	2967.13(8)	3042.7(12)	1706.4(3)	3897.8(13)	6377.7(7)	1929.81(9)
Z	2	4	2	1	2	4	2
ρ_{calc}g/cm^3	1.145	1.218	1.182	1.057	0.931	1.192	1.149
μ/mm^{-1}	0.994	1.058	1.031	0.918	0.804	0.99	0.827
F(000)	1156	1156	1156	578	1168	2424	712
Crystal size/mm^3	0.220 × 0.070 × 0.070	0.120 × 0.110 × 0.070	0.650 × 0.300 × 0.150	0.194 × 0.123 × 0.094	0.226 × 0.207 × 0.162	0.180 × 0.160 × 0.110	0.38 × 0.38 × 0.36
Radiation	MoKα (λ = 0.71073)	MoKα (λ = 0.71073)	MoKα (λ = 0.71073)	MoKα (λ = 0.71073)	MoKα (λ = 0.71073)	MoKα (λ = 0.71073)	MoKα (λ = 0.71073)
2Θ range for data collection/°	1.684 to 55.006	3.66 to 55.018	3.824 to 55.36	2.06 to 55.16	3.392 to 55.252	2.464 to 55.196	3.592 to 55.18
Index ranges	$-15 \le h \le 15$<br>$-15 \le k \le 15$<br>$-29 \le l \le 32$	$-15 \le h \le 15$<br>$-19 \le k \le 18$<br>$-19 \le l \le 22$	$-15 \le h \le 15$<br>$-21 \le k \le 21$<br>$-23 \le l \le 23$	$-8 \le h \le 9$<br>$-16 \le k \le 12$<br>$-24 \le l \le 24$	$-13 \le h \le 16$<br>$-21 \le k \le 25$<br>$-20 \le l \le 20$	$-28 \le h \le 28$<br>$-16 \le k \le 12$<br>$-29 \le l \le 29$	$-15 \le h \le 16$<br>$-16 \le k \le 16$<br>$-19 \le l \le 19$
Reflections collected	42821	27135	50541	28888	35804	54094	66156
Independent reflections	14245 [R_{int} = 0.0795 R_{sigma} = 0.1214]	6799 [R_{int} = 0.0452 R_{sigma} = 0.0550]	13902 [R_{int} = 0.1121 R_{sigma} = 0.1869]	7761 [R_{int} = 0.0259 R_{sigma} = 0.0332]	8928 [R_{int} = 0.0647 R_{sigma} = 0.0907]	14346 [R_{int} = 0.0976 R_{sigma} = 0.1050]	8857 [R_{int} = 0.0190 R_{sigma} = 0.0128]
Data/restraints/ parameters	14245/0/669	6799/0/335	13902/12/662	7761/0/335	8928/0/335	14346/0/705	8857/6/450
Goodness-of-fit on F^2	0.972	1.001	1.101	1.053	0.695	1.058	1.091
Final R indexes [I>=2σ (I)]	R_1 = 0.0550 wR_2 = 0.0935	R_1 = 0.0403 wR_2 = 0.0817	R_1 = 0.0533 wR_2 = 0.1410	R_1 = 0.0379 wR_2 = 0.0919	R_1 = 0.0615 wR_2 = 0.1798	R_1 = 0.0719 wR_2 = 0.1472	R_1 = 0.0400 wR_2 = 0.1054
Final R indexes [all data]	R_1 = 0.1398 wR_2 = 0.1228	R_1 = 0.0798 wR_2 = 0.0950	R_1 = 0.1479 wR_2 = 0.2051	R_1 = 0.0527 wR_2 = 0.0967	R_1 = 0.1353 wR_2 = 0.2266	R_1 = 0.1595 wR_2 = 0.2037	R_1 = 0.0490 wR_2 = 0.1149
Largest diff. peak/hole / e Å^3	0.46/-0.44	0.40/-0.47	1.57/-1.79	0.41/-0.32	1.12/-0.89	0.81/-1.37	0.97/-0.24

Table S2b Crystal data and structure refinements for **9-14**.

Identification code	**9**	**10**	**11**	**12**	**13**	**14**
CCDC number	2151196	2151197	2151198	2151199	2151200	2151201
Formula	$C_{64}H_{86}Ge_2N_4O_2Se_2$	$C_{64}H_{86}Cl_2Cu_2Ge_2N_4O_2$	$C_{64}H_{86}Br_2Cu_2Ge_2N_4O_2$	$C_{64}H_{86}Cu_2Ge_2I_2N_4O_2$	$C_{76}H_{86}Cu_4F_{10}Ge_2N_4O_3$	$C_{93}H_{126}Ge_3N_6O_3$
Formula weight	1246.46	1286.52	1375.44	1469.42	1692.82	1593.76
Temperature/K	296(2)	296(2)	293(2)	296(2)	296(2)	296.15
Crystal system	triclinic	monoclinic	monoclinic	triclinic	monoclinic	monoclinic
Space group	*P*-1	*P*2$_1$/*c*	*P*2$_1$/*c*	*P*-1	*P*2$_1$/*n*	*P*2$_1$/*c*
a/Å	13.0758(6)	12.2484(13)	12.22161(11)	12.736(7)	15.6334(4)	12.2624(4)
b/Å	13.2058(6)	17.0817(19)	17.25408(15)	13.448(7)	21.9405(6)	35.7163(12)
c/Å	21.8939(11)	37.929(4)	34.1247(3)	25.242(13)	24.1372(6)	25.1470(8)
α/°	102.931(2)	90	90	80.10(3)	90	90
β/°	101.420(2)	98.267(6)	93.6580(8)	78.07(3)	105.9780(10)	99.347(2)
γ/°	101.707(2)	90	90	74.18(3)	90	90
Volume/Å^3	3488.7(3)	7853.2(14)	7181.30(11)	4039(4)	7959.3(4)	10867.3(6)
Z	2	4	4	2	4	4
ρ_{calc}g/cm^3	1.187	1.088	1.272	1.208	1.413	0.974
μ/mm^{-1}	1.944	1.396	3.232	2.056	1.864	0.865
F(000)	1292	2680	2824	1484	3456	3384
Crystal size/mm^3	0.652 × 0.389 × 0.355	0.314 × 0.293 × 0.173	0.2 × 0.1 × 0.1	0.406 × 0.205 × 0.111	0.325 × 0.284 × 0.254	0.256 × 0.221 × 0.129
Radiation	MoKα (λ = 0.71073)	MoKα (λ = 0.71073)	CuKα (λ = 1.54184)	MoKα (λ = 0.71073)	MoKα (λ = 0.71073)	MoKα (λ = 0.71073)
2Θ range for data collection/°	3.29 to 55.066	2.62 to 55.014	5.19 to 154.314	3.372 to 50.498	2.554 to 54.968	1.998 to 50
Index ranges	-16 ≤ h ≤ 16<br>-17 ≤ k ≤ 17<br>-28 ≤ l ≤ 27	-14 ≤ h ≤ 15<br>-21 ≤ k ≤ 22<br>-47 ≤ l ≤ 49	-15 ≤ h ≤ 12<br>-21 ≤ k ≤ 19<br>-40 ≤ l ≤ 43	-15 ≤ h ≤ 15<br>-16 ≤ k ≤ 16<br>-30 ≤ l ≤ 29	-20 ≤ h ≤ 20<br>-24 ≤ k ≤ 28<br>-31 ≤ l ≤ 31	-13 ≤ h ≤ 14<br>-42 ≤ k ≤ 42<br>-29 ≤ l ≤ 29
Reflections collected	60767	69551	51995	45076	73533	243300
Independent reflections	16009 [R_{int} = 0.0281 R_{sigma} = 0.0317]	17701 [R_{int} = 0.0704 R_{sigma} = 0.0850]	14578 [R_{int} = 0.0533 R_{sigma} = 0.0518]	14084 [R_{int} = 0.0854 R_{sigma} = 0.0911]	18173 [R_{int} = 0.0339 R_{sigma} = 0.0459]	19124 [R_{int} = 0.0881 R_{sigma} = 0.0534]
Data/restraints/ parameters	16009/0/687	17701/0/705	14578/0/705	14084/0/705	18173/0/912	19124/0/953
Goodness-of-fit on F^2	1.027	0.996	1.053	1.056	0.989	1.09
Final R indexes [I>=2σ (I)]	R_1 = 0.0341 wR_2 = 0.0849	R_1 = 0.0519 wR_2 = 0.1065	R_1 = 0.0518 wR_2 = 0.1364	R_1 = 0.0760 wR_2 = 0.2024	R_1 = 0.0514 wR_2 = 0.1216	R_1 = 0.1481 wR_2 = 0.3422
Final R indexes [all data]	R_1 = 0.0545 wR_2 = 0.0906	R_1 = 0.1154 wR_2 = 0.1181	R_1 = 0.0661 wR_2 = 0.1418	R_1 = 0.1209 wR_2 = 0.2301	R_1 = 0.0922 wR_2 = 0.1355	R_1 = 0.1747 wR_2 = 0.3551
Largest diff. peak/hole / e Å^3	0.39/-0.26	0.48/-0.44	1.16/-0.99	1.24/-1.18	0.67/-0.70	1.70/-1.45
